# Untargeted In Silico Compound Classification—A Novel Metabolomics Method to Assess the Chemodiversity in Bryophytes

**DOI:** 10.3390/ijms22063251

**Published:** 2021-03-23

**Authors:** Kristian Peters, Gerd Balcke, Niklas Kleinenkuhnen, Hendrik Treutler, Steffen Neumann

**Affiliations:** 1 Bioinformatics & Scientific Data, Leibniz Institute of Plant Biochemistry, Weinberg 3, 06120 Halle (Saale), Germany; hendrik.treutler@gmail.com (H.T.); sneumann@ipb-halle.de (S.N.); 2 German Centre for Integrative Biodiversity Research (iDiv) Halle-Jena-Leipzig, Deutscher Platz 5e, 04103 Leipzig, Germany; 3 Institute of Biology/Geobotany and Botanical Garden, Martin Luther University Halle-Wittenberg, 06108 Halle (Saale), Germany; 4 Cell and Metabolic Biology, Leibniz Institute of Plant Biochemistry, Weinberg 3, 06120 Halle (Saale), Germany; gbalcke@ipb-halle.de; 5 Max Planck Research Group Chromatin and Ageing, Max Planck Institute for Biology of Ageing, Joseph-Stelzmann-Str. 9b, 50931 Cologne, Germany; NKleinenkuhnen@age.mpg.de; 6 MS-Platform, Cluster of Excellence on Plant Sciences, Botanical Institute (CEPLAS), University of Cologne, 50931 Cologne, Germany; 7 Datameer GmbH, Magdeburger Straße 23, 06112 Halle (Saale), Germany

**Keywords:** compound classes, classification, chemical ecology, metabolomics, chemodiversity, biodiversity, chemical traits, mosses, bryophytes, data-independent acquisition

## Abstract

In plant ecology, biochemical analyses of bryophytes and vascular plants are often conducted on dried herbarium specimen as species typically grow in distant and inaccessible locations. Here, we present an automated in silico compound classification framework to annotate metabolites using an untargeted data independent acquisition (DIA)–LC/MS–QToF-sequential windowed acquisition of all theoretical fragment ion mass spectra (SWATH) ecometabolomics analytical method. We perform a comparative investigation of the chemical diversity at the global level and the composition of metabolite families in ten different species of bryophytes using fresh samples collected on-site and dried specimen stored in a herbarium for half a year. Shannon and Pielou’s diversity indices, hierarchical clustering analysis (HCA), sparse partial least squares discriminant analysis (sPLS-DA), distance-based redundancy analysis (dbRDA), ANOVA with post-hoc Tukey honestly significant difference (HSD) test, and the Fisher’s exact test were used to determine differences in the richness and composition of metabolite families, with regard to herbarium conditions, ecological characteristics, and species. We functionally annotated metabolite families to biochemical processes related to the structural integrity of membranes and cell walls (proto-lignin, glycerophospholipids, carbohydrates), chemical defense (polyphenols, steroids), reactive oxygen species (ROS) protection (alkaloids, amino acids, flavonoids), nutrition (nitrogen- and phosphate-containing glycerophospholipids), and photosynthesis. Changes in the composition of metabolite families also explained variance related to ecological functioning like physiological adaptations of bryophytes to dry environments (proteins, peptides, flavonoids, terpenes), light availability (flavonoids, terpenes, carbohydrates), temperature (flavonoids), and biotic interactions (steroids, terpenes). The results from this study allow to construct chemical traits that can be attributed to biogeochemistry, habitat conditions, environmental changes and biotic interactions. Our classification framework accelerates the complex annotation process in metabolomics and can be used to simplify biochemical patterns. We show that compound classification is a powerful tool that allows to explore relationships in both molecular biology by “zooming in” and in ecology by “zooming out”. The insights revealed by our framework allow to construct new research hypotheses and to enable detailed follow-up studies.

## 1. Introduction

The identification of chemical constituents underlying specific ecological and molecular functioning has become a powerful tool in plant biology [1,2,3]. It has enabled us to pinpoint compounds that exert specific bioactivities, play vital roles in molecular pathways, are produced as adaptations to environmental or climatic changes, or describe molecular interactions with other organisms [4]. For several decades, the technology of metabolomics has been applied, e.g., LC/MS now allows the detection of the majority of semi-polar compounds in plant samples [5]. However, it is only recently that metabolomics has been applied in ecology [1,2]. In ecological metabolomics, metabolite fingerprinting strategies are most often applied, where characteristic sets and subsets of metabolite features such as compounds, fragments, and adducts are related to ecological functioning either within plants at molecular scales or at more coarse ecological scales without specifically targeting some molecules [4]. With untargeted ecometabolomics, the emphasis is now shifting from finding metabolite fingerprints underlying a specific functioning to a more detailed investigation of metabolites at broader levels [6].

In the field of ecometabolomics, typically non-model species are studied that have important functions in ecosystems but those species are biochemically not well characterized. Despite the fact that only approximately 24,000 species of bryophytes are known today [7], they have evolved a high diversity of bioactive compounds to interact with the environment and with other organisms. As they quickly respond to environmental changes and pollution to sustain their homeostasis, bryophytes are considered distinctive bioindicators [8,9,10] and can be keystone components in some ecosystems [11]. For instance, bryophytes often colonize early successional stages and by collecting debris, storing water and solidifying soil they pave the way for other organisms [12]. Bryophyte cushions can act as nurseries for a variety of larval stages of insects and serve as indirect food reserves for birds and insect feeding animals [13,14]. Bryophytic species interact with the environment and other organisms by producing a large diversity of “infochemicals” that mediate various kinds of ecological functioning [15]. For example, male plants of the mosses *Bryum argenteum* and *Ceratodon purpureus* were found to emit volatile compounds that attract micro-arthropods, such as springtails and mites that may transport sperm released by the mosses antheridia to neighboring female plants, enhancing sporophyte production [16,17]. To date, the mechanistic functions, including genetic factors, of the majority of these volatile compounds and other secondary metabolites have not been explored in detail [18,19].

In plant ecology, experiments are often conducted in the field, which may involve the sampling of species in remote and inaccessible locations where it is often impossible to carry the equipment to shock-freeze plant material in order to preserve it for later biochemical analyses. As a result, plant material is collected and transported in ambient conditions, and eventually stored in herbarium collections. This is especially true for bryophytes, where the majority of specimens are collected in herbaria as species determination often requires subsequent microscopic investigation. When bryophytes are stored as herbarium specimen, they undergo physiological changes which can be described as the combined effects of drought, oxidative stress, light inhibition, and apoptosis. While some liverworts, such as the genus *Scapania* lose their oil bodies in just a few minutes or hours when drying, for the bryophytes *Tortula muralis* and *Riccia macrocarpa* it has been reported that they are able to survive prolonged periods of drought and sustain desiccation and low relative humidity for 10–20 years [20,21,22]. While desiccation tolerance is a widespread trait in bryophytes, only few vascular species (approximately 70 fern and 60 angiosperms) are known that can withstand similar conditions [23,24]. Physiologically, upon dehydration species like liverworts of the genus *Scapania* experience a breakdown of cellular membranes, such as those surrounding the oil bodies. By contrast, desiccation-tolerant bryophytes possess protective mechanisms and produce specific compounds in order to maintain cellular integrity during both de- and re-hydration [24,25]. For instance, some bryophytes, such as *Polytrichum* spp., produce hydroxylated cinnamic acids that form lignin-like structures (“proto-lignin”) in conducting cell wall tissues that protect them from dehydration and reinforce cellular integrity [26,27]. Proto-lignin-like compounds have not been found so far in liverworts such as *Scapania* which may explain their susceptibility to desiccation. Physiological changes under herbarium conditions also include oxidative stress. Bryophytes have been found to produce a variety of compounds like flavonoids, anthocyanins, alkaloids, terpenes, (bis)bibenzyls covering a large diversity of compound families that protect them from reactive oxygen species (ROS) [28,29]. As the biochemical adaptations are very complex, little is known about the role of particular secondary metabolites and corresponding genes so far [18,30,31].

Recently, technological advances in high resolution LC/MS–MS-based metabolomics enabled the quantitative assessment of signals obtained from MS1 and simultaneous acquisition of associated MS/MS spectra. While MS1 peak intensities are typically used to compare the levels of secondary metabolites between groups, MS/MS fragment spectra often allow to obtain insights into structural information of the respective analytes. With different strategies, techniques such as data dependent acquisition (DDA) and data independent acquisition (DIA) rapidly trigger collision-induced dissociation allowing the collection of thousands of MS/MS spectra in one chromatographic analysis [32]. Both techniques first perform a full MS1 scan. In DDA, the intensity of the MS1 precursors is ranked to fragment only the most abundant precursor ions, which can bias the analysis as lower abundant signals remain unfragmented. The precursor ions and the acquired fragmented spectra are inherently linked, resulting in monoisotopically pure mass spectra. By contrast, DIA partitions all MS1 precursors in the full mass range in predefined isolation windows and acquires multiple MS/MS fragment spectra irrespective of the ion intensity [33]. Mass and retention time related features are extracted by specialized software such as MS-Dial [34] and XCMS [35]. As the readouts are multiplexed in DIA, direct links between the precursor ions and the fragment spectra are missing. This mandates subsequent chromatographic signal deconvolution and increases the complexity of the bioinformatic processing to increase spectral purity [36,37]. Studies comparing DDA and DIA approaches revealed considerably higher spectral coverage for DIA [38].

Recent improvements allow to extract thousands to a few ten thousands of MS1 features with their associated MS/MS spectra from a single chromatographic run [39]. However, typically 90% or more of the spectral metabolomics data in ecometabolomics studies contain compounds that never had been characterized before. This includes so called “known unknowns” (molecules with undefined structure that have been measured before) and “unknown unknowns” (molecules for which neither their identity nor their chemical structure is known) [40,41]. This is particular true for non-model organisms that are biochemically not well characterized, due to few analytical standards available, and very sparse spectral annotations in reference libraries [42]. Exploring the multitude of unknown compounds (the so called “dark matter” [43]) remains a significant challenge as the *de novo* structure elucidation and the identification is a very elaborate and a time-consuming process and often requires the use of additional analytical measurements. Lately, approaches have emerged that are performed in silico which can predict chemical structures based on molecular fingerprints or fragmentation patterns via computational methods [44,45]. However, they can only provide putative structures for a comparably small number of metabolites.

A viable alternative is the annotation of mass spectrometric features at the level of metabolite families. As the complexity and information of metabolic profiles can be overwhelming, studies with ecological context are often aiming at a more global description of metabolic patterns—a method called metabolite fingerprinting [46]. Even though compound classification provides “only” a generalized identity instead of explaining molecular identity in detail [47,48,49], in ecology this abstracted information can be more powerful.

Formerly, the determination of compound classes required the use of targeted analytical methods. Although there are classical extraction-based, chromatographic, electrophoretic, and structure-indicative spectroscopic methods (i.e., NMR) available, applying these methods often take biochemists months of work to characterize the different sets of compound classes separately and mandate extensive purification of individual compounds [50]. Some of these methods may also be coupled to a mass spectrometer, but only allow for targeted analyses [51].

In the last few years, advances in computing power and the availability of libraries with a sufficient amount of spectral information made it possible to perform classification in silico using computational methods. These are much faster than traditional biochemical methods but their accuracy greatly depend on the quality of the acquired MS/MS fragment spectra and on the used algorithms. The development of a chemical ontology and the tool ClassyFire, which identifies compound classes from molecules with known structure, greatly facilitated these efforts [52,53]. There are numerous approaches described, such as MS2LDA, which decomposes fragment data to annotate molecular substructures [54], ClassyFirePredict, which can classify chemical motifs from spectral data of the Global Natural Products Social Molecular Networking platform (GNPS) using an artificial neural network [48], GNPS molecular networking, which is a community-based effort to annotate structural libraries [55], MetFamily, which performs hierarchical clustering on fragment spectra and allows annotation of different clusters of metabolite families [47], compMS2Miner, which calculates a consensus score for compound classes using external software [56], MS-Dial using a method to assign spectra to chemical motifs [36], a hybrid approach combining CSI:FingerID with ClassyFire [57], and CANOPUS, which can classify unknown fragment spectra using a deep neural network [49]. Even though many different compound classification algorithms have been developed, a general framework is still missing on how to apply these approaches to specific research questions.

Traditionally, the fields of molecular biology and ecology operate at distinct spatiotemporal scales with different resolution [4]. Metabolomics is a key technology that cannot only be applied in both of these contrasting research fields, but also used to connect these fields by providing a comprehensive view on any systemic organismal process without prior knowledge [49]. However, in molecular biology a systemic overview on molecular networks can be extremely complex, whereas in ecology the complexity of metabolite patterns can be overwhelming. Compound classification is an ideal tool to simplify these overly complex molecular networks and metabolite patterns. In molecular biology, discriminating compound classes are first determined and broadly assigned to molecular traits. In an additional step, by focusing only on the relevant compound classes, molecular entities are assigned to specific molecular networks while simultaneously accelerating the annotation processes. This approach allows to “zoom in” and further mechanistically relate compounds or compound classes to specific cellular and molecular processes [58]. By contrast, in ecology, compound classification allows for comparative approaches of different species, for relating ecological traits to species’ biochemistry, or for exploring the responses of species to different conditions [6]. This is being achieved by “zooming out”—relating patterns in compounds or compound classes to ecological functioning at more coarse scales using multivariate statistics such as ordination methods [4].

In this study, we present a general methodological framework to comprehensively assess the chemical diversity using in silico classification. We apply the MetFamily classifier (available at https://github.com/ipb-halle/MetFamily, accessed on 22 March 2021), which employs a machine learning approach on consensus spectral information of overrepresented fragmentation patterns [42,47]. While our preceding study [42] has focused on the classification of the most abundant compound classes present in bryophytes via DDA, in this study we apply the new data annotation methodology using untargeted raw DIA–MS/MS data from LC– sequential windowed acquisition of all theoretical fragment ion mass spectra (SWATH)–QToF analyses, which captures the majority of semi-polar compounds in biological samples. To demonstrate the general applicability of our framework, we use this representative data set with bryophytes, which is openly available in the repository MetaboLights as MTBLS851 [59].

Here, we specifically target research questions in chemodiversity and molecular ecology. We investigate metabolite profiles of ten different species of bryophytes and provide a detailed comparative analysis at the level of compound classes comparing samples, which were collected in a fresh state (shock-frozen on-site as it would be in the lab) and stored in an herbarium for half a year. We show that our untargeted in silico classification framework provides an elaborate overview on broad systemic changes. The insights revealed by our framework enable a more detailed understanding of mechanistic molecular processes and allow to construct new research hypotheses and detailed follow-up studies.

## 2. Results

Raw metabolite profiles including the MS1 metabolite fingerprinting tables, MS/MS fragment spectra acquired in DIA mode, classification tables, and corresponding data tables, classifiers and meta-data are available as identifier MTBLS851 in the repository MetaboLights [59]. Additional results from metabolite fingerprinting, a comparison between positive and negative ion modes, quality control plots and tables of the MetFamily classification performances are available in the Supplementary Material. Abbreviations of species names used in the plots and the text are described in the section Abbreviations.

### 2.1. Chemical Diversity Analyses

**Figure 1:**
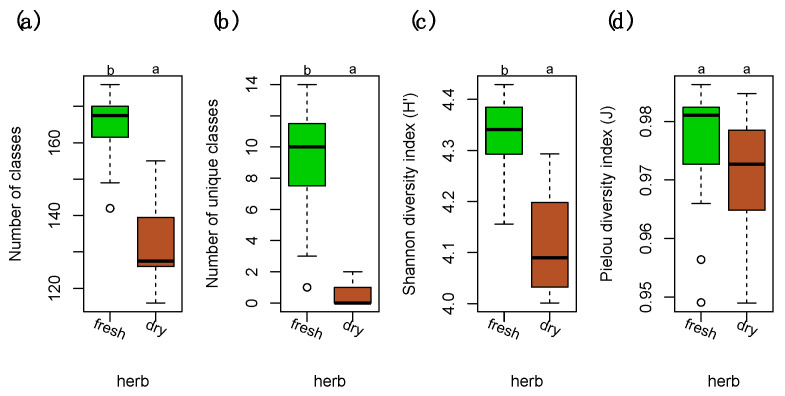
Diversity measures of compound classes in fresh and dry herbarium conditions. (**a**) Number of total compound classes. (**b**) Number of unique compound classes that were exclusively present in either of the levels. (**c**) Shannon diversity indices (*H’*) for the relative richness of compound classes present in fresh vs. dry. (**d**) Pielou’s evenness (J) for the homogeneity of the distribution of the numbers of compound in the classes. Differences among groups (letters on the top of the plot) were calculated with performing the Tukey honestly significant difference (HSD) test post hoc on a one-way ANOVA. Different letters show significant differences (*p* < 0.05). n = 40 for each level. Factor levels were colored: green for fresh and brown for herbarium conditions.

The chemodiversity was investigated at the level of compound classes using our in silico classification approach which was applied to samples acquired in positive and negative ion mode separately. The data matrices were joined afterwards into a single matrix. Both the number of compounds (chemical richness) and the number of compounds unique to either of the levels was significantly larger in fresh samples than in samples stored in herbarium conditions (Figure 1a,b). This resulted in a significantly larger Shannon diversity index for fresh samples (Figure 1c). The Pielou’s evenness was relatively similar in fresh and herbarium samples (Figure 1d).

**Figure 2:**
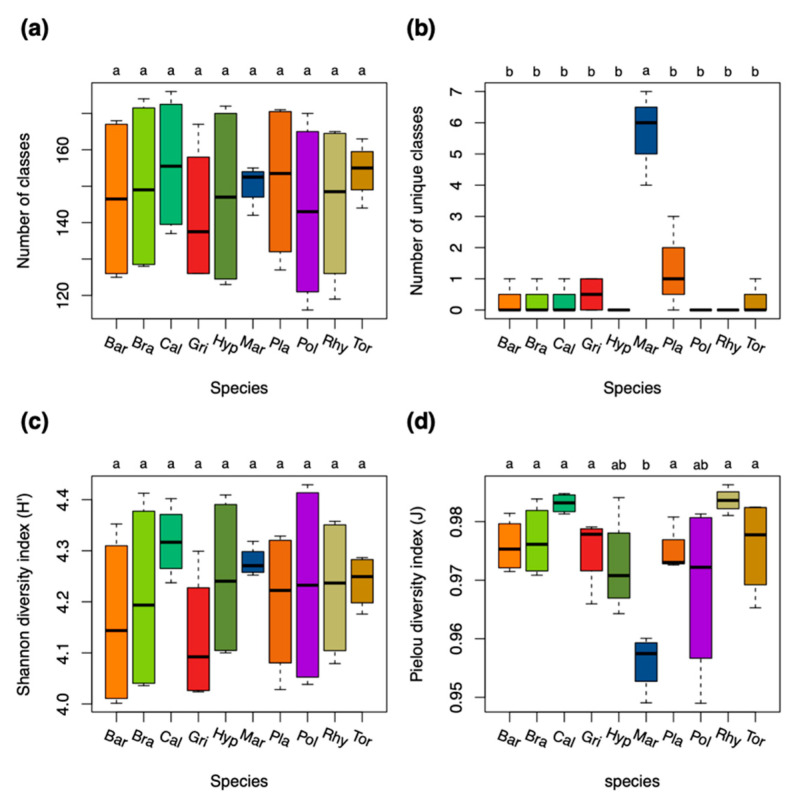
Diversity measures of compound classes for the ten bryophyte species. (**a**) Number of total compound classes. (**b**) Number of unique compound classes that were exclusively present in either of the species. (**c**) Shannon diversity indices (*H’*) for the relative richness of compound classes present in the species. (**d**) Pielou’s evenness (J) for the homogeneity of the distribution of compound classes in the species. Differences among groups (letters on the top of the plot) were calculated with performing the Tukey HSD post hoc on a one-way ANOVA. Different letters show significant differences (*p* < 0.05). n = 8 for each species. Species were colored: red and brown colors for acrocarpous species, green and yellow colors for pleurocarpous species, and blue color for liverworts. Species codes are explained in the Abbreviations section.

Diversity analyses at the species level showed that *Marchantia polymorpha (Mar*) had significantly more unique compound classes than the other species (Figure 2b). The homogeneity in the richness of compound classes was significantly lower in *Mar* when compared to the other species due to significantly more compounds in the dry condition (Figure 2d). The diversity was otherwise relatively similar in both acrocarpous and pleurocarpous species (Figure 2a–d).

### 2.2. Exploring Differences in the Composition of Metabolite Families

**Figure 3:**
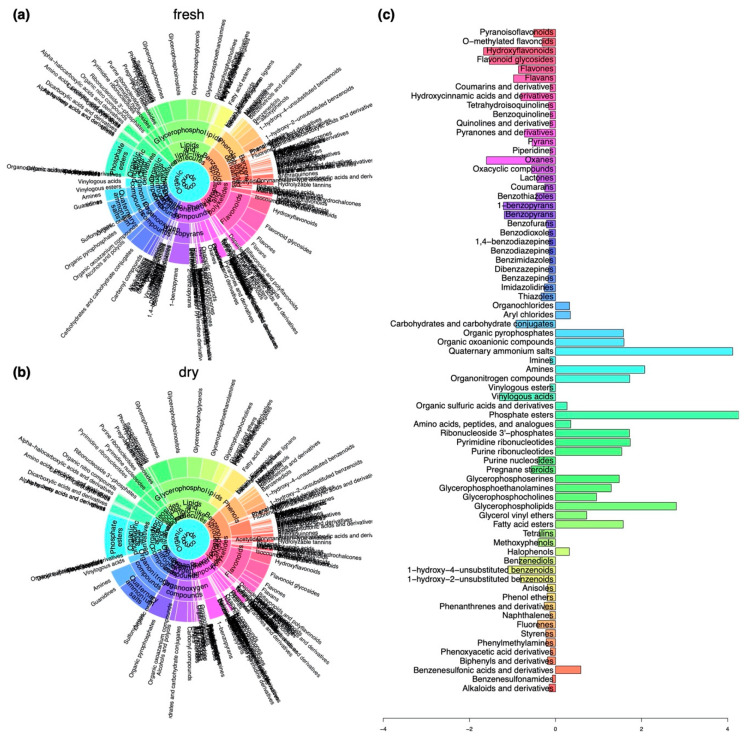
Differences in the composition of compounds belonging to compound classes at different levels of the chemical ontology with regard to herbarium conditions. (**a**) Sunburst plot showing the composition of compounds in samples that were collected in a fresh state. (**b**) Sunburst plot showing the composition of compounds in samples that were stored in the herbarium. (**c**) Changes in relative frequencies of compound classes when comparing fresh with herbarium samples determined using the Fisher’s exact test. Negative values indicate a decline and positive values mark an enrichment in the respective compound classes under herbarium conditions.

Differences in the composition of compound classes comparing fresh and dry herbarium conditions were explored using sunburst plots (Figure 3a,b) and the Fisher’s exact test (Figure 3c). In the herbarium samples we found an increase in compounds belonging to the superclass of organic acids derivatives (compounds belonging to phosphate esters); organic nitrogen compounds (compounds belonging to quaternary ammonium salts, amines and organonitrogen compounds); the superclass of lipids and lipid-like molecules (several glycerophosphorylated compound classes like glycerophospholipids, -serines, -ethanolamines, and -cholines, fatty acid esters); organic oxygen compounds (compounds belonging to organic pyrophosphates, organic oxoanionic compounds); nucleosides, nucleotides, and analogues (compounds belonging to ribonucleotides including purine and pyrimidine nucleotides and nucleosides); and benzenesulfonic acids and derivatives (Figure 3).

We determined decreases in the numbers of compounds present in the superclasses of organoheterocyclic compounds (oxanes, pyrans, pyranones, and derivatives, oxacyclic compounds, benzopyrans, benzothiazoles, benzodiazepines, (di)benzazepines, benzofurans, carbohydrates, and carbohydrate conjugates, thiazoles, lactones), benzenoids (benzenediols, unsubstituted benzenoids, styrenes, tetralins, methoxyphenols), phenylpropanoids and polyketides (hydroxyflavonoids, flavonoid glycosides, flavans, flavones, pyranoisoflavonoids, hydroxycinnamic acids and derivatives, coumarins and coumarins and their derivatives), organic acids and derivatives (vinylogous acids), and lipids and lipid-like molecules (pregnane steroids) (Figure 3).

**Figure 4:**
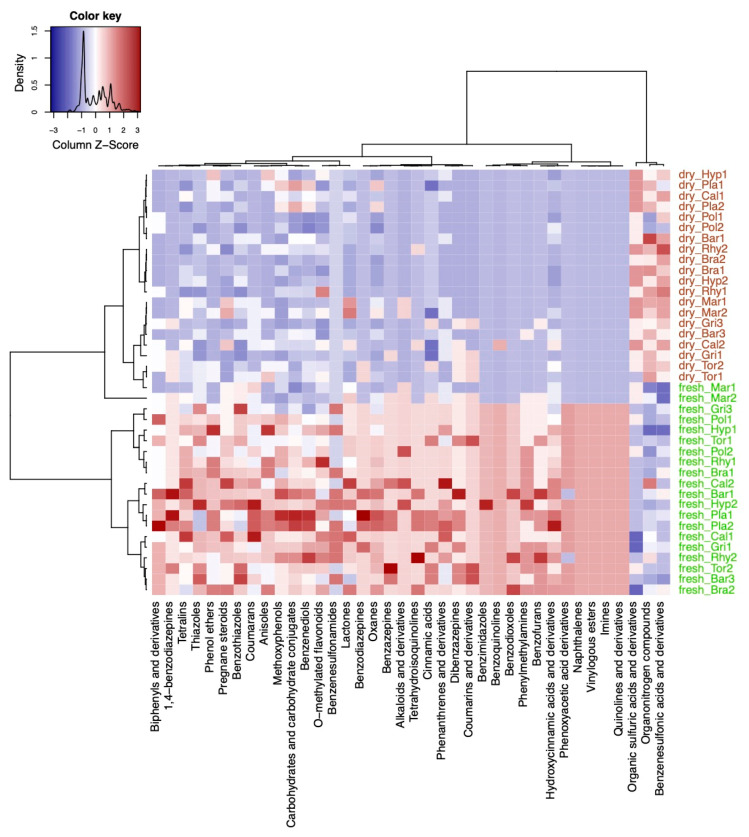
Results from the variable selection with sparse partial least squares discriminant analysis (sPLS-DA). Heatmap of the selected variables by the sPLS-DA model using the compound classification table. Compound classes that were enriched are shown in red color and classes that were decreased are shown in blue color in the plots. Sample names starting with either fresh or dry indicate fresh and herbarium samples, respectively. The species codes are further explained in the abbreviations section. Factor levels in the column were colored: green for fresh and brown for herbarium conditions.

A variable selection using sparse partial least squares discriminant analysis (sPLS–DA) was performed to evaluate how the differences in compound classes between fresh and dry conditions were constituted in the different species (Figure 4). In short, the multivariate sPLS–DA found similar results as the univariate Fisher’s exact test (Figure 3c). From the selected compound classes, fresh samples of *Mar* were clustered together with the herbarium samples of the other species (Figure 4). Only the classes of alkaloids and derivatives, pregnane steroids, benzothiazoles, and lactones were selected that were more present in fresh vs. herbarium samples in *Mar* (Figure 4). *Plagiomnium undulatum (Pla*) and *Polytrichum strictum (Pol*) were more dissimilar with regard to richness in compound classes when compared to the other species.

sPLS-DA selected the classes of organic sulfuric acids, organonitrogen compounds, and benzenesulfonic acids and derivatives to be significantly enriched in herbarium samples except for *M. polymorpha* (Figure 4).

**Figure 5:**
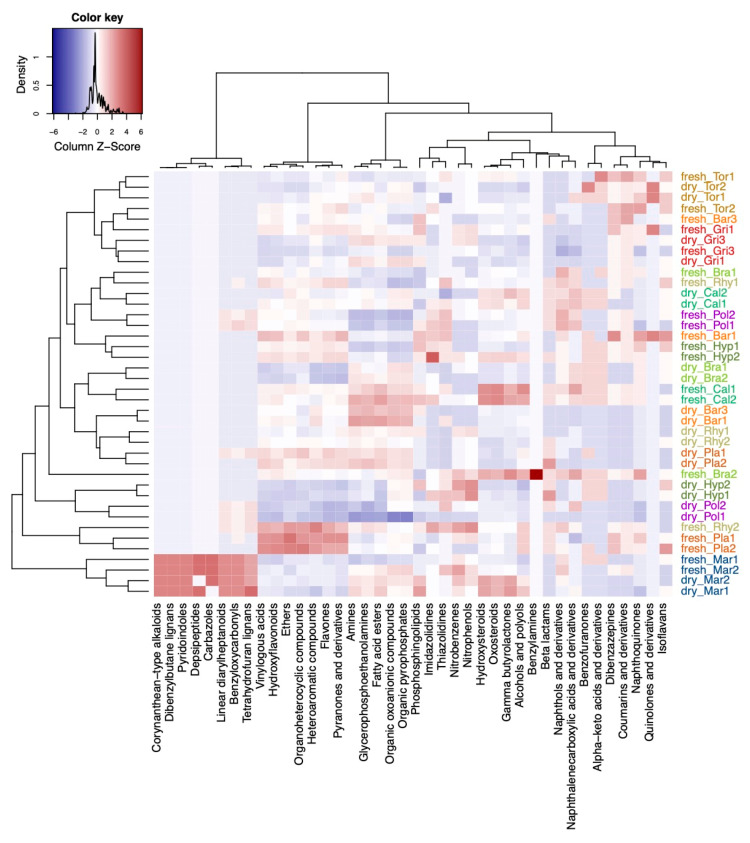
Differences in the composition of compounds belonging to compound classes at different levels of the chemical ontology with regard to the different species. Shown is a heatmap of the selected variables by the sPLS-DA model. Compound classes that were enriched in the species are shown in red color and classes that were decreased are shown in blue color in the plots. Sample names starting with either fresh or dry indicate fresh and herbarium samples, respectively. The species codes are further explained in the Abbreviations section. Species were colored: red and brown colors for acrocarpous species, green and yellow colors for pleurocarpous species, and blue color for liverworts.

Next, we compared differences in numbers of compound classes in the different species (Figure 5). The sPLS-DA model selected five compound classes which were unique for *Mar*: corynanthean–type alkaloids, dibenzylbutane lignans, pyridoindoles, depsipeptides, carbazoles (Figure 5). The compound classes of linear diarylheptanoids, benzyloxycarbonyls, and tetrahydrofuran lignans were also found in *Pla* and *Pol* and *Rhy* (*Rhytidiadelphus squarrosus*). The compound classes of vinylogous acids, hydroxyflavonoids, ethers, organoheterocyclic compounds, heteroaromatic compounds, flavones, and pyranones and derivatives were enriched in fresh and dry samples of *Pla*, and in fresh samples of the pleurocarpous species *Rhy*, *Hyp* (*Hypnum cupressiforme*), *Bra* (*Brachythecium rutabulum*), and *Cal* (*Calliergonella cuspidata*). Hydroxysteroids, oxosteroids, gamma butyrolactones, alcohols and polyols were enriched in *Cal* (fresh and dry) and *Bra* (fresh only), and *Mar* (fresh only) when compared to the other species. Compounds belonging to beta lactams, naphthols and derivatives, and naphthalenecarboxylic acids and derivatives were enriched in pleurocarpous species. The acrocarpous species were less distinct than liverworts and pleurocarpous species and rather characterized by relatively low numbers in the aforementioned compound classes. *Pol* had enriched phosphosphingolipids, imidazolidines, and thiazolidines when compared to other acrocarpous species. In general, dibenzazepines, coumarins and derivatives, and isoflavans were more likely associated with acrocarpous species (Figure 5).

### 2.3. Comparison of Chemotaxonomic Analysis with Phylogeny

**Figure 6:**
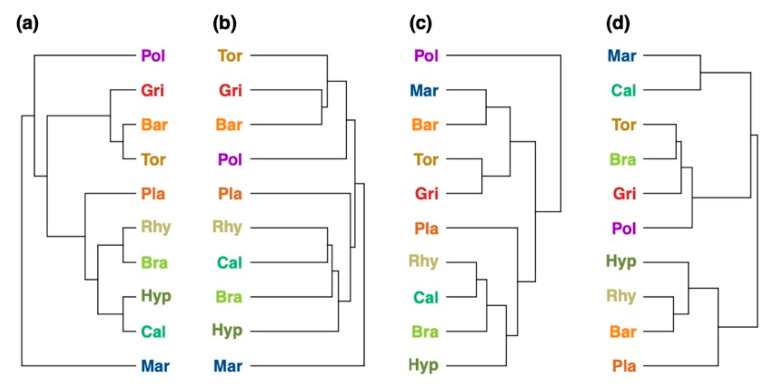
Comparison of the (**a**) phylogenetic tree with the (**b**–**d**) chemotaxonomic trees of the 10 bryophyte species reconstructed by (**b**) metabolite fingerprinting, (**c**) in silico classification performed in negative ion mode, and (**d**) in silico classification performed in positive ion mode. The phylogenetic tree showing the phylogenetic relationships of the ten bryophyte species. Comparison metrics: (**b**) Mantel statistic: r = 0.321, cophenetic correlation coefficient: c = 0.653, Robinson–Foulds metric: rf = 0.5; (**c**) Mantel statistic: r = 0.005, cophenetic correlation coefficient: c = 0.042, Robinson–Foulds metric: rf = 0.75; (**d**) Mantel statistic: r = 0.151, cophenetic correlation coefficient: c = 0.0, Robinson–Foulds metric: rf = 1.0. Species names were colored: red and brown colors for acrocarpous species, green and yellow colors for pleurocarpous species, and blue color for liverworts. The species codes are explained in the Abbreviations section.

A phylogenetic tree of the bryophytes was extracted from the Open Tree of Life project (Figure 6a) and compared with chemotaxonomic trees reconstructed from the metabolite fingerprinting data (Figure 6b), compound classification data in negative ion mode (Figure 6c), and compound classification data in positive ion mode (Figure 6d). The chemotaxonomic information from metabolite fingerprinting allowed a good reconstruction of phylogenetic relationships (Figure 6a). Liverworts, pleurocarpous and acrocarpous bryophytes were clustered in separate clades, with *Mar* being the most dissimilar species. The chemotaxonomic clustering of the in silico classification data in negative ion mode (Figure 6c) resulted in *Pol* to be the most dissimilar species with regard to the richness and composition of its compound classes. Acrocarpous and pleurocarpous species were clustered apart from each other. In contrast to the phylogenetic tree, *Mar* was clustered together with the acrocarpous species. The chemotaxonomic tree calculated from the classification data in positive ion mode (Figure 6d) resulted in a heterogeneous distribution of species in the tree which was in contrast to the phylogenetic tree. *Mar* was still the most dissimilar species, but acrocarpous and pleurocarpous species were intermixed.

### 2.4. Trait Analysis

**Figure 7:**
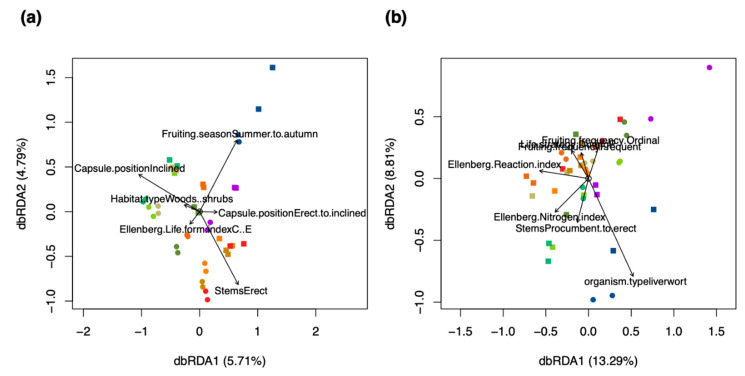
Relationships of ecological characteristics with (**a**) the metabolite fingerprinting data, and (**b**) in silico classification data. The lengths of the arrows represent the explanatory power of the variable with regard to the data. The positions of the samples relative to the direction of the arrow represent the relationship of the samples with the variable. The scores were colored according to the ten species used in the study: red and brown colors for acrocarpous species, green and yellow colors for pleurocarpous species, and a blue color for liverworts. A round shape of the scores represents samples of dry herbarium conditions and a square represents samples of fresh conditions. n = 80.

Relationships of ecological characteristics were investigated using distance-based redundancy analysis (dbRDA) with regard to the metabolite fingerprinting and in silico classification data. Ecological characteristics like Ellenberg indices and capsule positions were acquired from literature for the used bryophyte species. Tables are available in [60,61]. The dbRDA model using the metabolite fingerprinting data explained a variation of 10.54% on the first two axes of the RDA ordination (Figure 7a). The model found that the metabolism of pleurocarpous species was associated with inclined capsule positions, procumbent stems, plagiotropic growth form, and relatively large spore diameters (a mean of 18.75 µm for pleurocarpous vs. 15.1 µm for acrocarpous species). The acrocarpous *Pla* was clustered near the pleurocarpous species due to having similarly sized spores (28 µm large). Metabolite features of acrocarpous species were associated with erect to inclined capsule positions, and those of *Mar* were separated by the characteristic vegetative regeneration via gemmae (Figure 7a).

Investigating the dbRDA comparing the in silico classification data with ecological characteristics revealed a model that explained greater variation (21.86%) than metabolite fingerprinting on the feature level (10.54%) (Figure 7a,b). The composition of compound classes in the different species was found to be related to environmental characteristics like the Ellenberg moisture, reaction and nitrogen indices (Figure 7b). The discrimination of species was larger in the metabolite fingerprinting data, where also species-specific characteristics like growth forms and capsule positions were more important than environmental characteristics (Figure 7a,b).

## 3. Discussion

The annotation of fragment spectra in LC/MS–MS data is still a highly challenging task [62]. Annotation is typically performed by searching spectra in spectral libraries, such as MassBank, GNPS, or the WEIZMASS library [55,63,64,65]; or by using in silico annotation tools like MetFrag, CFM-ID or SIRIUS [44,66,67]. However, metabolite structure databases usually cover only few compounds present in samples of biological non-model species. A viable alternative are in silico classifiers that perform a less specific approach and avoid complex biochemical preparation procedures [54,68,69]. In this study, we used the MetFamily classifier, which recognizes structural features in fragment spectra instead of predicting the structure of entire molecules. The classifier has been trained on sets of usable high-quality spectra from the MassBank of North America (MoNA) database and is currently representing the 677 compound classes covered by MoNA [63].

Chemical classification can be performed at different levels of the chemical ontology [53]. When interpreting the results from the MetFamily classifier, it returns a ranked list of candidate classes per spectrum similarly to the results of in silico annotation tools like MetFrag or SIRIUS [44,66]. The correct class may not be ranked first and could be hidden in the list of alternative parents. For instance, a flavonoid glycoside could primarily be classified as a glycoside, or a diterpenoid primarily as a phenylpropanoid. Both classifications are correct but may not reflect the desired classification level from a biochemist’s perspective. Here, interpretation of the results can be facilitated when restricting the classification to a specific level of the chemical ontology, i.e., at the level of superclasses, classes or subclasses; which is still meaningful in ecometabolomics. The results of our classifier can be improved by increasing the number of high-quality spectra for training the machine learning model covering a wider range of compound classes. The scoring algorithm of the classifier can also be adapted by integrating skilled biochemists in the classification process. Furthermore, methods, such as deep learning and boosted regression trees, are promising to improve the accuracy of the model [49]. However, the classification of truly novel substances will remain a challenge due to missing reference spectra.

Our chemical classification framework revealed several insights into the chemodiversity of bryophytes and molecular changes under herbarium conditions. The lower chemodiversity of metabolite profiles show that many secondary metabolites degrade due to breakdown. The compound classes of quaternary ammonium salts, amines, and other organonitrogen compounds were enriched in herbarium samples indicating the degradation of amino acids, peptides, and analogues as products of a non-functional respiration metabolism and general catabolic processes [70,71,72]. The classes of quaternary ammonium salts are also structurally related to many functional proteins and nitrogen containing glycerophospholipids, which constitute metabolites produced by bryophytes and lichens against desiccation and as a response to reactive oxygen species [28,73]. We further detected an increase in phosphate esters and nucleosides, nucleotides and analogues, which are likely involved in phosphorylation in the fatty-acid metabolism in order to retain membrane structure [31].

It has often been described that bryophytes produce carbohydrates that protect them from osmotic stress and desiccation; thus, carbohydrates are involved in the glycolysis and gluconeogenesis, the synthesis of proto-lignin to stabilize cell walls, the citrate cycle, and photosynthesis and related sugar metabolisms [74,75]. Our results show a downregulation of carbohydrates under herbarium conditions, which suggests that carbohydrates detected by LC/MS only play minor roles with protection from desiccation and degradation [73].

Flavonoids have been reported to be one of the most diverse groups of natural products with regard to the response of bryophytes to environmental changes, including the protection from excessive light, temperature and drought stress, and various biotic interactions [76,77]. When comparing fresh with herbarium samples, we detected significantly fewer flavonoids (including the subclasses hydroxyflavonoids, flavonoid glycosides, flavones, flavans, and hydrocinnamic acids). The decrease can be explained by the generally low stability of flavonoids. Under severe stress, bryophytes may further stimulate the degradation of flavonoids to scavenge free radicals [78]. As flavonoids have been described to regulate protein stability, their absence would also explain the many quaternary ammonium salts and fragments of amino-acids under herbarium conditions.

In the herbarium samples, we detected the absence of many steroids and their derivatives. They perform important roles in plant development, are precursors in the synthesis of phytohormones, and are involved in defenses against pathogens and in the degradation of xenobiotics (e.g., pollution) [9,79,80,81]. In *Marchantia*, bibenzyls and bisbybenzyls are the most abundant steroidal products. They are considered important biomarkers with regard to biotic and environmental interactions and the development of pharmaceutical and bio-control agents [18,28,29,82]. Under herbarium conditions, many of these highly bioactive products degrade or fragment into smaller components. We further detected that many benzenic compounds were lost from herbarium samples. Bryophytes produce structurally complex benzenoids, including various phenolics, lignans, phenylpropanoids, and terpenes. Many of these compounds have been found to be located in the oil bodies of liverworts [83], explaining the functional roles related to biotic and abiotic stress responses in living tissues [84,85].

In ecometabolomics, metabolite fingerprinting is now widely used to reveal metabolic patterns that drive ecological functioning. Here, we present a computational framework to annotate metabolite families using DIA–LC/MS–MS data. We were able to assign MS/MS fragment spectra to 91.5–95.7% of MS1 features (in negative and positive ion mode, respectively) and, thus, capturing the predominant part of semi-polar metabolites in plant samples. Our analytical methodology allows for a near complete picture of the composition of secondary metabolites in the tested bryophyte species. Despite the fact that classification simplifies information on the characteristics of compounds, the results from our ordination with dbRDA show that when correlating ecological characteristics with the classification data, the level of explained variance is improving (Figure 7). However, as the intra-specific variability of species was very large, environmental responses of bryophytes explained more variance in the composition of compound classes than species-specific and life-history characteristics. This is likely due to the fact that bryophytes produce a magnitude of secondary metabolites in order to maintain homeostasis to balance for a lack of specialized anatomical structures in response to environmental conditions [10]. Our results are in line with other findings that bryophytes produce low amounts of nitrogen-containing compounds, which has been attributed to an evolutionary advantage in nitrogen-poor environments [10,86,87]. Here, bryophytes restrict nitrogen-containing products to structurally complex metabolites like benzenoids (phenolics), phenylpropanoids (terpenes), amino acids, and alkaloids [70,88].

As with many ecometabolomics studies, the amount of molecular information can be overwhelming. Our results show that classification can be used to improve and facilitate the ecological interpretation of the physiological and biochemical responses of bryophytes to biotic and abiotic environmental conditions [6]. Patterns in the diversity of metabolite families and related physiological characteristics can be used to define chemical traits that further explain variations in species distributions and niches [25], which are described by a close relationship between bryophyte functional categories and microhabitat conditions [89]. The trait analyses from this study also suggest that indicator values and certain life-history characteristics are not suited to correlate molecular responses of bryophytes to environmental conditions. We suggest to use a combination of morphological and anatomical traits, such as shoot mass per area, photosynthetic nitrogen use efficiency, specific stem diameter, height, or leaf area, which have been described to perform better for vascular plants [87,90].

We propose to use our in silico classification framework in future studies of ecometabolomics to evaluate chemical traits (to evaluate those compound classes that have major ecological impact or serve as bioindicators for certain ecological relationships). For instance, using the flavonoid richness as a chemical trait to quantitatively assess the exposition to sunlight of the sampled individuals in the habitat instead of the generalized Ellenberg indicator for light, which is an average literature value for the species. We expect that when using these novel chemical traits, ecological explanation can be improved [91,92,93]. Further research and detailed comparative studies are needed to precisely assess the composition and richness of metabolite families in bryophytes and other plants. As in many ecometabolomics studies DDA–LC/MS–MS is already part of the analytical pipeline, our classification framework can be directly applied to these data and, thus, enabling new insights through the evaluation of chemical traits based on metabolite families. Our framework can also be applied retroactively to existing data sets, where the full extent of DDA–LC/MS–MS may have not been explored at the global level and, thus, fostering the re-use of ecometabolomics data [94].

Chemotaxonomic analyses have been considered to be a viable alternative over complicated DNA sequencing of bryophytes due to the lack of detailed genetic maps and the presence of repetitive sequences [85]. We encourage to use our framework to explore inter-specific variability in the composition of metabolite families of species to differentiate phylogenetically relevant metabolites or compound classes in different species (“phylomarkers”) that can be used to determine cryptic species without requiring expensive DNA barcoding methods [85,95,96]. The presence or absence of particular biomarkers or compound classes has become an important tool for the identification of lichens and has been applied for almost 50 years [96,97,98,99,100]. Comparing the chemical diversity of different bryophyte species may also shed some more light on the chemical evolution of phylogenetically closely (i.e., cryptic species) or distantly related species, may reveal previously unknown connections in molecular networks, and can accelerate the finding of phylogenetically distinct biomarkers [95]. The results from this study showed that reconstructing the chemotaxonomic tree from classification data in negative ion mode was more consistent with phylogeny than when using classification data in positive ion mode. The results from the trait analysis indicate that in negative ion mode more species-specific metabolites were acquired by our analytical setup, whereas more metabolites regarding environmental adaptation (e.g., correlations of compound classes to Ellenberg Reaction index) were acquired in positive ion mode, which highlights the need to acquire both ionization modes in experiments.

In plant ecology, biochemical analyses are often performed on herbarium plant material as experiments are often conducted in the field. The results from this study show that the spectrum of compounds found in herbarium material was considerably dissimilar to fresh material. As a result, chemodiversity analyses conducted solely on herbarium specimen may be biased and the validity of results may be restricted to the classes which are found in herbarium samples. Our results show that the differences between fresh and herbarium samples are highly species-specific. Further research is needed to explore how large the spectrum of compound classes is changed in different species when sampled fresh vs. using herbarium specimen. Once the changes in the composition of compound classes in different species have been determined, regression methods like nonlinear local polynomial (LOESS) based normalization or support vector regression (SVR) may be applied in order to correct for the bias [101,102,103].

Our novel classification framework can provide a broad overview on the chemical characteristics of any kind of biological species by assessing the chemodiversity and the composition of compound classes. By “zooming out” biochemical patterns can be related to ecological functioning at more coarse scales. As most bryophytes are typically non-model species, they represent an ideal use case in the research field of ecometabolomics as they contain a large portion of completely novel compounds. Our method can be applied at different levels of the chemical ontology, i.e., at the level of superclasses, classes or subclasses, potentially revealing ecological patterns at different scales. We also kept our framework flexible so that it is possible to use different spectral classifiers (e.g., among others CFM-ID, GNPS, CANOPUS [49,57,67]).

We further recommend to apply our framework in classic metabolomics studies. For instance, it is well known that plants produce more flavonoids when grown in sunlight compared to growing in shade. Automatically selecting the relevant compounds belonging to the chemical class of flavonoids would greatly facilitate the work of biochemists as they could focus the identification only on the selected spectra of a particular metabolite family. In comparative studies, classification would also allow to directly investigate the role of particular metabolite families, helping to characterize physiological processes more quickly at broader levels, to uncover patterns in metabolite fingerprints, and to simplify complex molecular patterns in order to find associated and expressed genes [58]. Thus, our classification framework is perfectly suited for hypotheses generation and to create follow-up studies by focusing on particular compounds or compound classes.

## 4. Materials and Methods

### 4.1. Sampling

Samples of the ten bryophyte species Bar (Barbula unguiculata Hedw.), Bra (Brachythecium rutabulum (Hedw.) Schimp.), Cal (Calliergonella cuspidata (Hedw.) Loeske), Gri (Grimmia pulvinata (Hedw.) Sm.), Hyp (Hypnum cupressiforme Hedw.), Mar (Marchantia polymorpha L.), Pla (Plagiomnium undulatum (Hedw.) T.J. Kop.), Pol (Polytrichum strictum Menzies ex Brid.), Rhy (Rhytidiadelphus squarrosus (Hedw.) Warnst.), and Tor (Tortula muralis Hedw.) were collected at random locations in the area of the Botanical Gardens of the Martin Luther University of Halle-Wittenberg. Sampling was performed in spring on 18 May 2017at stable and sunny conditions after mid-day between 1:00 p.m. and 3:00 p.m.

Four composite samples of the sterile gametophytes (multiple individuals) of each species were taken, leading to a total of 4 × 10 = 40 samples. Only above-ground parts of gametophytes, such as leaves, branches, stems, or thalloid parts were taken for sampling. Visible archegonial and antheridial heads and any belowground parts such as rhizoids and rooting stems were removed with a sterile tweezer. One half of the sample collection (2 × 10 = 20 samples) were put in Eppendorf tubes and instantly frozen on dry ice. The other remaining fresh samples (2 × 10 = 20 samples) were put in non-chlorinated tea bags (to allow air-drying and to avoid possible chemical side-reactions with the tea bags) and stored in an herbarium at Leibniz Institute of Plant Biochemistry. On 14 November 2017, two composite samples of each herbarium-dried species (2 × 10 = 20 samples) were put in Eppendorf tubes and stored at −80 °C. On 30 January 2018, samples were prepared for the analytical analysis. Metabolomics analyses were performed based on protocols of [39,104]. They are described in detail further below.

### 4.2. Metabolite Extraction

A total of 25 mg of dry and 50 mg fresh tissue were weighed under cryogenic conditions into wall-reinforced cryo-tubes of 1.6 mL volume (Precellys Steel Kit 2.8 mm, Peqlab Biotechnologie GmbH, Erlangen, Germany) filled with 3 steel beads (3 mm), 1 steel bead (5 mm), and 200 mg glass beads. Then, 900 µL of a cold mixture of dichloromethane/ethanol (−80 °C) was added first followed by 150 µL of 30 mM HCl (4 °C). Cell rupture/metabolite extraction was assisted by FastPrep bead beating (3 × 20 s, speed 6.5 m/s, FastPrep24, MP Biomedicals LLC, Santa Ana, CA, USA) followed by phase separation by centrifugation at 20,000× *g* (2 min, 4 °C). After removal of the upper aqueous phase, 100 µL 30 mM HCl were added and bead milling and centrifugation were repeated. The aqueous phase was completely removed.

Semi-polar and apolar metabolites were collected from the denser organic phase, from which the extract was aspirated from the bottom using a narrow bore pipet. Exhaustive extraction was achieved by addition of 500 μL fresh tetrahydrofuran (THF) to the cell debris, bead milling, and centrifugation. The second organic extract was aspirated from top, unified with the aliquot of the first organic extract and dried in a stream of nitrogen gas before storage at −80 °C. Semi-polar metabolites dissolved in 180 µL 80% MeOH were centrifuged and the supernatant was collected for LC/MS analysis within 24 h.

### 4.3. Metabolite Separation

Separation of medium polar metabolites was performed on a Nucleoshell RP18 (2.1 × 150 mm, particle size 2.1 µm, Macherey & Nagel, GmbH, Düren, Germany) using a Waters ACQUITY UPLC System, equipped with an ACQUITY Binary Solvent Manager and ACQUITY Sample Manager (20 µL sample loop, partial loop injection mode, 5 µL injection volume, Waters GmbH, Eschborn, Germany). Eluents A and B were aqueous 0.3 mmol/L NH_4_HCOO (adjusted to pH 3.5 with formic acid) and acetonitrile, respectively. Elution was performed isocratically for 2 min at 5% eluent B, from 2 to 19 min with linear gradient to 95% B, from 19–21 min isocratically at 95% B, and from 21.01 min to 24 min at 5% B. The flow rate was set to 400 µL/min and the column temperature was maintained at 40 °C. Metabolites were detected by positive and negative electrospray ionization and mass spectrometry. The analytical Quality Control (QC) involved batches with QC-standard mixtures (“MM10”) and blank samples to control memory effects and to ensure retention time stability. The MM10 mixture included the following compounds which give peaks in negative and positive ion modes: DL-a-Phenylglycine (151.06333), 6-Furfurylaminopurine (215.08071), Rutin (610.15339), o-Anisic acid (152.04734), Phlorizin Dihydrat (436.13695), 3-Indolylacetonitrile (156.06875), N-(3-Indolylacetyl)-L-valine (274.13174), Biochanin A (284.06847), Carnosol (330.18311), Abietic acid (302.22458). Additionally, the column was washed with 2-propanol to remove also hydrophobic residues after each batch. Afterwards the column was washed back with 100% acetonitrile for column storage.

### 4.4. Untargeted Mass Spectrometry

Untargeted mass spectrometric analysis of small molecules was performed with MS–ToF–SWATH–MS/MS (TripleToF 5600, both Sciex GmbH, Darmstadt, Germany) operating in negative or positive ion mode and controlled by Analyst 1.6 TF software (Sciex GmbH, Darmstadt, Germany). The source operation parameters were as following: ion spray voltage, −4500 V/+5500 V; nebulizing gas, 60 psi; source temperature, 600 °C; drying gas, 70 psi; curtain gas, 35 psi. TripleToF instrument tuning and internal mass calibration were performed every 5 samples with the calibrant delivery system applying APCI negative or positive tuning solution, respectively (Sciex GmbH, Darmstadt, Germany).

For the MS1 measurements, ToF masses were scanned between 65 and 1250 Dalton with an accumulation time of 50 ms and a Q2 energy of 10 V (−10 V for negative ion mode), which is the vendor recommended setting for improved ion transmission in MS1 [39]. The MS2-SWATH-experiments were divided into segments of 20 ms accumulation time. Together the SWATH experiments covered the entire mass range from 65 to 1250 Dalton in 48 separate scan experiments, which allowed a cycle time of 1.1 s. As chromatographic peak widths varied depending on concentration and analyte, the core shell technology was used, which normally produces peaks in the range of very few seconds (<20 s). For the narrowest peaks 6–10 duty cycles per peak were managed. For an average peak at least 10 cycles per peak were achieved with our instrument [104]. Throughout all MS/MS scans a declustering potential of 35 (or −35 V) was applied. Collision energies for all SWATH–MS/MS were set to 35 V (−35) and a collision energy spread of ±25 V, maximum sensitivity scanning, and elsewise default instrument settings [104].

### 4.5. Raw Data Acquisition

Centroid raw data were converted to the mzML format with the msconvert tool of the ProteoWizard software suite [105]. The converted raw data were uploaded to MetaboLights and metadata were recorded in compliance with the minimum information guidelines for Metabolomics studies [106,107]. Profiles in positive and negative ion mode were recorded and used for further data analyses.

Prior to data analyses, a matrix with two factors was constructed containing the levels for herbarium (fresh, dry) and the species (Bar, Bra, Cal, Gri, Hyp, Mar, Pla, Pol, Rhy, Tor).

### 4.6. Peak Detection

Chromatographic peak detection was performed in R 4.0.2 (available at https://cran.r-project.org, accessed on 8 January 2021) with the package XCMS 3.11.7 [35] separately for positive and negative ion modes. The following settings were used performing the centWave algorithm: ppm = 25, mzCenterFun = mean, prefilter = c(3, 100), peakwidth = c(10, 20), snthresh = 5, integrate = 2. Chromatographic peaks were then grouped using the factor matrix as pheno-data. Grouping was based on time dimension peak densities using PeakDensityParam with the parameters: minFraction = 0.5 and bw = 20. Retention time correction was performed using the parameters minFraction = 0.5, smooth = loess, span = 0.2 and family = Gaussian. Peaks were grouped again afterwards. In order to assess the performance of the peak detection, a technical validation procedure (quality control) was performed. Deviations in *m*/*z* and retention times were evaluated in the metabolite profiles of the bryophytes and the MM10 samples as part of the analytical quality control. Figures S11–S15 in the Supplementary Material show these quality control plots.

Peak detection of MS/MS peaks in all isolation windows (buckets) of the DIA-mode was used with the same parameters as the MS1 peak detection using the findChromPeaksIsolationWindow function of XCMS. Only MS/MS spectra with precursors between retention times of 60 to 1200 s were retained (using the filterRT function). Peaks before 60 s would contain numerous salt signals and non-retained hydrophilic metabolites, which were too difficult to separate without exact retention time information. Late eluting peaks (>1200 s) contained lipids with very broad elution profiles and were therefore excluded as well. For each MS1-level peak the according MS/MS spectra were reconstructed using the function reconstructChromPeakSpectra with the parameter minCor = 0.5. The MS/MS spectral information was converted to a peak list using the fData function.

The MS1-level peak table was constructed with the function featureValues and the parameters method = medret and value = into were applied. Only those peaks were kept for which MS/MS spectral information were reconstructed (91.5% for negative mode, 95.7% for positive mode). Overlapping spectra were merged using the function combineSpectra using the parameters mzd = 0.05, ppm = 50, and intensityFun = “max”. All spectra present in a sample were saved in MSP files, which were used later for the in silico classification. The peak table was log transformed and missing values were imputed with zeros. A binary matrix to be used for compositional analyses was created containing the presence and absence values of compounds. A compound was deemed as present when its abundance was above 0.1% the abundance of the largest peak.

The above steps were implemented for positive and negative ion mode separately. The data matrices were joined afterwards into a single matrix.

### 4.7. In Silico Classification

In silico classification was performed using the MetFamily classifier [42,47] for positive and negative ion modes separately. The MetFamily classifier was created using a machine learning approach on a training set of approximately 57,000 MS/MS fragment spectra in negative and positive ion modes with known structures from the MassBank of North America (MoNA) [63] enriched with terms from the ChemOnt ontology (version 2.1) [53]. In a first step, the training data were cleaned in such a way that only fragment spectra of usable quality are being used for training. This step resulted in a total of 11,328 fragment spectra for negative ion mode and 21,908 for positive ion mode. The MetFamily algorithm first decomposed the spectral information of the spectra into neutral losses. Then, an internal fragment matrix was constructed containing one row for each compound and one column for each neutral loss as described in [47]. The resulting sparse fragment matrix was assembled from the consensus spectra with an absolute *m*/*z* error of 0.01 Dalton and a relative *m*/*z* error of 20 ppm. In order to guarantee a minimum level of representativeness, metabolite families were restricted to (1) contain at least ten different fragment spectra; (2) selected subtrees must have at least one fragment present in at least 75% of the spectra within the cluster; (3) each cluster must contain at least five compounds; and (4) when criterion 2 was not fulfilled for all other clusters which contained the current cluster. Consensus spectra were learned from the common features of all spectra belonging to the remaining metabolite families (where each metabolite family was equivalent to a compound class as defined by a unique ChemOnt term and represented by a particular cluster in the hierarchical clustering tree). Finally, a binary classifier was constructed for each of the metabolite families. A *p*-value was calculated using the background scores distribution in accordance to [47]. A stratified repeated subset validation procedure was employed with ten data set compositions with 70% training data set and 30% test data set. The MetFamily classifier obtained consensus spectra for a total of 363 compound classes in negative mode and 451 compound classes in positive mode.

When the learned MetFamily classifier is applied to an unknown spectrum, it provides scores for the primary compound class and scores for alternative parents in case it detected spectral features, which are also present in other classes. The usage and methodology is further explained further in [42,47].

The MetFamily classifier was applied on any spectra present in each sample with the following parameters: msms.intensity.threshold = 10, minimumIntensityOfMaximalMS2peak = 0.01%, minimumProportionOfMS2peaks = 0.05, mzDeviationAbsolute_grouping = 0.01, mzDeviationInPPM_grouping = 25, doPrecursorDeisotoping = TRUE, mzDeviationAbsolute_precursorDeisotoping = 0.001, mzDeviationInPPM_precursorDeisotoping = 10, maximumRtDifference = 0.02, doMs2PeakGroupDeisotoping = TRUE, mzDeviationAbsolute_ms2PeakGroupDeisotoping = 0.01, mzDeviationInPPM_ms2PeakGroupDeisotoping = 10, proportionOfMatchingPeaks_ms2PeakGroupDeisotoping = 0.9, mzDeviationAbsolute_mapping = 0.01, minimumNumberOfMS2PeaksPerGroup = 1, neutralLossesPrecursorToFragments = TRUE, neutralLossesFragmentsToFragments = FALSE. The classification scores of primary compound class and alternative parents were saved for all compounds present in samples in separate matrices for positive and negative ion modes. Tables containing the classification performances on the metabolite families present in the samples are available in the Supplementary Material as Tables S1 and S2. For the subsequent analyses, only the information on the primary compound class were used.

### 4.8. Statistical and Diversity Analyses

Statistical analyses were carried out with R 4.0.2 and the following additional packages: BiocParallel, doParallel, RColorBrewer, multtest, MSnbase, vegan, multcomp, Hmisc, gplots, lme4, lmerTest, glmnet, VennDiagram, dtwclust, pROC, PRROC, mltest, multiROC, ape, dendextend, cba and phangorn. Prior to the statistical analyses, peak and classification tables were log transformed to reach a semi-normal distribution. Missing values were imputed with zeros. No other normalization on the data were performed.

To get an overview on the influence of study factors and separation of samples, Principal Component Analysis (PCA) was carried out using the function prcomp. Variance partitioning from the package vegan (function varpart) was calculated for the study factors species and herbarium to evaluate the amount of variation explained in the data tables for the study factors [108]. Variable selection of metabolite features that were related to either particular species’ or herbarium conditions was performed using Sparse Partial Least Squares Discriminant Analysis (sPLS-DA) using the function splsda from the mixOmics package [109]. As with discriminant analysis the number of components relate to the number of interactions of factor levels and the explained variations of the factor levels scatter on the corresponding component axes, the parameter ncomp was chosen 2 for herbarium, and 10 for species, respectively. A vector of variables to keep (parameter keepx) was constructed using the amount of variation explained from the variance partitioning multiplied by the number of total metabolite features in the data table. A heatmap was constructed with the selected variables using the function cim of the package mixOmics and variables extracted that correspond to the levels of either species or herbarium. In order to evaluate the performance of the fitted model, M-fold cross-validation with 10 folds using the function perf was performed and the following additional performance measures were calculated: multi-class area under curve (AUC) (function ml_test of the package mltest), the R^2^ of the fitted vs. the entire model, and Receiver Operating Characteristic (ROC) and PR (Precision and Recall) curves using the functions plot.roc and ci.se from the pROC package and the function pr.curve from the PRROC package [110,111,112,113]. The area under curve (AUC) was calculated from the ROC and the area under precision recall curve (AUC-PR) from the PR curve, respectively. Summaries of model performances are available in Supplementary Material.

Diversity analyses were applied on the data of the metabolite fingerprinting (peak table) and on the compositional data of the classification approach (classification table). Analyses were performed calculating the compound richness (sum of compounds per each sample), the Shannon diversity index (*H’*) on compounds in each sample and the Pielou’s evenness (*J*) based on the *H’* divided by the log transform of the richness [114,115]. The following Equation (1) was used to calculate the Shannon diversity index *H’*:

(1) $$H^{\prime }=\;\overset{t}{\underset{i=1}{\sum }}p_{i}\;\ln\left(p_{i}\right)$$

The Pielou’s evenness *J* was calculated according to Equation (2):

(2) $$J=\;\frac{H^{\prime }}{\log S}$$

where the relative species/compound abundance *p_i_* is calculated as following: pi= NiN; *N_i_* is the abundance of the *i*-th species/compound and N is the total abundance of all species/compounds; and S is the species richness. These diversity measures can be used as sample sizes were equal between levels [116].

Unique compounds were determined when a compound was present only in one species and not the others, and for fresh vs. dry, respectively. To compare abundances between the different species and the herbarium conditions, boxplots were generated using the function boxplot in R. The Tukey honestly significant difference (HSD) test on a one-way ANOVA was performed post hoc using the multcomp package to test the factor levels for significant differences.

Sunburst plots were constructed from the compositional data tables mainly using the functions draw.sector and arctext from the packages circlize, plotrix, and gplots [117]. These plots show an overview of the richness of compounds with regard to the ontology of the corresponding compound classes. In order to compare sunburst plots and find significant differences, the Fisher’s exact test was used creating contingency tables for all compound classes separately using the function fisher.test in R [118,119]. *p*-values below 0.005 were treated as significantly. A simple barplot was constructed for the significant classes showing the enrichment or reduction of their relative abundances. The frequency of the relative abundances *f* was calculated using Equation (3), where *k* is the respective compound class and *n* the total number of classified compound classes:

(3) $$f_{k}=\frac{dry_{k}}{\sum_{i=1}^{n}dry_{i}}-\frac{fresh_{k}}{\sum_{i=1}^{n}fresh_{i}}$$

A phylogenetic tree based on taxonomic information of bryophytes was extracted from the Open Tree of Life project with the identifier opentree12.3@mrcaott541ott1066 [120]. The supertree was pruned to only contain phylogenetic information for the used species in the study using the R package ape. Dendrograms using the chemotaxonomic information of the feature and the classification tables were created separately by constructing a cophenetic distance matrix using Bray-Curtis dissimilarity and hierarchical clustering was performed using Ward.D as agglomeration method. The two topmost nodes containing the acrocarpous and pleurocarpous groups in the dendrograms were swabbed using the function reorder of the package dendextend to improve visual representation. In order to compare the phylogenetic tree with the chemotaxonomic information of the metabolite fingerprinting and the classification, Procrustes analysis and the Mantel test (r) were performed together with calculating Pearson’s correlation (c) and the Robinson–Foulds metric (rf).

In order to investigate the relation of ecological characteristics with the peak and classification tables, distance-based redundancy analysis (dbRDA) using the function dbrda of the package vegan was carried out. The ecological characteristics described in [60,61] were used. Bray-Curtis was chosen as distance measure. Ordinal and categorical ecological characteristics were transformed to the presence–absence matrices for the ordination. Significant characteristics were chosen using the function ordistep with forward variable selection. The model performance was evaluated using the goodness of fit statistic (squared correlation coefficient) by applying the function envfit post-hoc on the dbRDA ordination. Similarly, using the envfit function, arrows were fitted on the ordination plot where the length of the arrow is proportional to the correlation between species ordination and the respective characteristics.

### 4.9. Data Records

Raw data and meta-data were uploaded to the data repository MetaboLights and are available under the identifier MTBLS851. The vignettes and data to recreate the plots are available in MetaboLights (https://www.ebi.ac.uk/metabolights/MTBLS851, accessed on 22 March 2021) and in Zenodo (https://zenodo.org/record/4627092, doi: 10.5281/zenodo.4627092, accessed on 22 March 2021).

## Data Availability

The raw data and meta-data presented in this study are openly available in MetaboLights (https://www.ebi.ac.uk/metabolights/MTBLS851). The vignettes and data to recreate the plots are available in Zenodo (https://zenodo.org/record/4627092, doi: 10.5281/zenodo.4627092).

## Supplementary Materials

Supplementary Materials can be found at https://www.mdpi.com/1422-0067/22/6/3251/s1.

## Abbreviations

acrocarpous	Phylogenetic clade of bryophytes having mostly an erect stem and sporophytes originate at the top of stems
ANOVA	Analysis of Variance
AUC	Area Under receiver operating characteristic Curve
AUC-PR	Area Under Precision Recall Curve
Bar	Species code for *Barbula unguiculata* Hedw., ott1027526
Bra	Species code for *Brachythecium rutabulum* (Hedw.) Schimp., ott734784
Bryophytina	Subdivision of plants on which the phylogenetic tree was constructed, ott471195
Cal	Species code for *Calliergonella cuspidata* (Hedw.) Loeske, ott405418
dbRDA	Distance-based Redundancy Analysis
DIA	Data Independent Acquisition
dry	Factor level: Samples which were taken under herbarium conditions
fresh	Factor level: Samples which were taken under fresh conditions
Gri	Species code for *Grimmia pulvinata* (Hedw.) Sm., ott604969
Hyp	Species code for *Hypnum cupressiforme* Hedw., ott160023
LC/MS	Liquid chromatography coupled to a Mass Spectrometer
Mar	Species code for *Marchantia polymorpha* L., ott56596
MS1	Mass-spectral analysis
MS/MS	Tandem mass-spectral analysis where two MS are performed resulting in fragment spectra
ott	Phylogenetic identifier for species and phylogenetic groups
PCA	Principal Component Analysis
Pla	Species code for *Plagiomnium undulatum* (Hedw.) T.J. Kop., ott744339
pleurocarpous	Phylogenetic clade of bryophytes having mostly a branched prostrate stem and sporophytes are produced in branches
Pol	Species code for *Polytrichum strictum* Menzies ex Brid., ott101288
PR	Precision and Recall
R	R statistical programming language
R^2^	R-squared
Rhy	Species code for *Rhytidiadelphus squarrosus* (Hedw.) Warnst., ott734791
ROC	Receiver Operating Characteristic
sPLS-DA	Sparse Partial Least Squares Discriminant Analysis
SWATH	Sequential Windowed Acquisition of All Theoretical Fragment Ion Mass Spectra
Tor	Species code for *Tortula muralis* Hedw., ott318814
Tukey HSD	Honestly significant difference test
XCMS	Framework to perform detection of chromatographic peaks
