# Supplementary material for: Untargeted In Silico Compound Classification—A Novel Metabolomics Method to Assess the Chemodiversity in Bryophytes
Source: Int J Mol Sci. 2021 Mar 23;22(6):3251. doi: 10.3390/ijms22063251 (PMC8005083; doi:10.3390/ijms22063251)
Supplement: Supplementary file 1 [file ijms-22-03251-s001.pdf]

Supplemental Material

Here, we present some additional results to the main manuscript. The results presented in the Supplemental Material constitute traditional metabolite fingerprinting performed at the MS1-level, including a comparison between positive and negative ion modes. Further, plots such as ROC and AUC-PR curves and model performances of the MetFamily classifier are presented.

S1. Results

S1.1. Metabolite Fingerprinting

Detection of chromatographic peaks at MS1 level was performed separately for negative and positive ion modes. 2798 metabolite features were present in the negative ion mode, and 5667 features for positive mode. PCA was performed on data tables for both modes, and the explained variance was similar (negative mode: PC1=28.2%, PC2=6.52%; positive mode: PC1=29.8%, PC2=4.62%) (Fig. 1). Separation on the PC1-axes of both modes corresponded to the herbarium condition and separation on PC2-axes was largely due to variation in the species in the metabolite profiles.

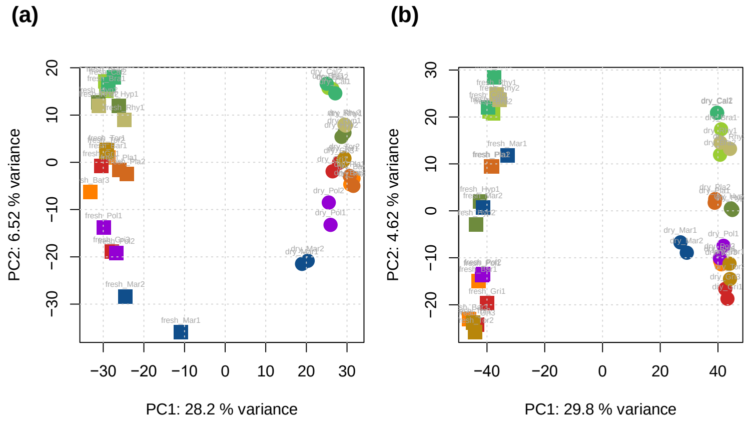

Figure S1. Principal Component Analysis of extracted chromatographic peaks in **(a)** negative ion mode, and **(b)** positive ion mode. The scores were colored according to the 10 species used in the study: red and brown colors for acrocarpous species, green and yellow colors for pleurocarpous species, and blue color for liverworts. A round shape of the scores represents samples of dry herbarium conditions and a square represents samples of fresh conditions. Separation on the PC1-axes of both modes corresponded to the herbarium condition and separation on PC2 was largely due to the species.

Next, selection of metabolite features that correspond to the herbarium conditions (fresh vs. dry) was performed using variable selection with sPLS-DA in negative and positive ion modes separately (Fig. 2). The sPLS-DA found the majority of metabolite features to be significantly related to the herbarium conditions of which the approx. 2/3 of the selected features were more abundant in the herbarium condition in both modes (Fig. 2a,b).

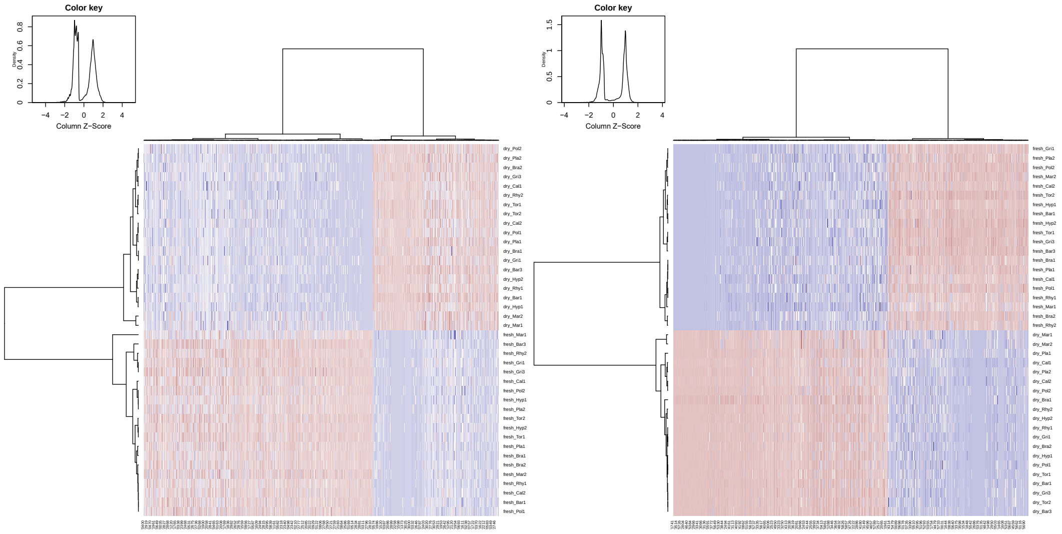

Figure S2. Heatmaps of the sPLS-DA with variable selection for the herbarium conditions (fresh vs. dry) on the peak tables in **(a)** negative ion mode, and **(b)** positive ion mode. Metabolite features that were enriched under dry conditions are shown in red color and features that were decreased are shown in blue color in the plots. Model performances: (a) AUC=1.0; (b) AUC=1.0.

Selection of metabolite features that correspond to the 10 bryophyte species was performed using variable selection with sPLS-DA in negative and positive ion modes (Fig. 3). The sPLS-DA found a total of 322 features in negative mode and 233 features in positive mode that were significantly related to the species (Fig. 3a,b).

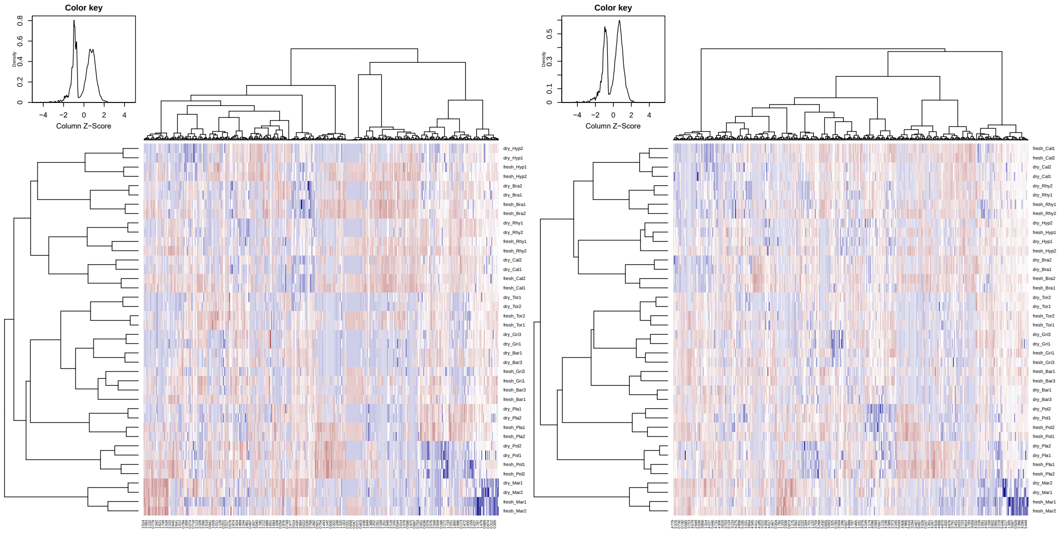

Figure S3. Heatmaps of the sPLS-DA with variable selection on the peak tables **(a)** negative ion mode, and **(b)** positive ion mode. Metabolite features that were enriched under dry conditions are shown in red color and features that were decreased are shown in blue color in the plots. Model performances: (a) Multiclass-AUC=0.775, AUC-PR: 0.996; (b) Multiclass-AUC=0.6975, AUC-PR=0.984.

S1.2 Diversity analysis on metabolite fingerprinting data

Diversity was investigated at MS1 level using the peak table containing relative abundances of metabolite features from negative and positive ion modes. There were no significant differences in the total number of metabolite features and in the number of unique features in fresh and dry samples (Fig. 4a,b). As expected, we found significantly fewer metabolite features (lower H') with larger abundances (larger J) when samples were stored in the herbarium when compared to fresh conditions (Fig. 4c,d).

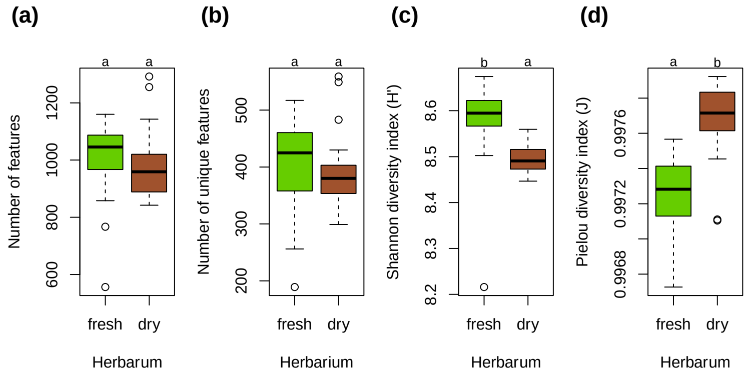

Figure S4. Diversity measures of metabolite features in fresh vs. herbarium conditions. **(a)** Number of total features. **(b)** Number of unique features that were exclusively present in either of the levels. **(c)** Shannon diversity indices (H') for the relative richness of features present in fresh vs. dry. **(d)** Pielou's evenness (J) for the homogeneity of the distribution of relative abundances. Differences among groups (letters on the top of the plot) were calculated with performing the Tukey HSD post hoc on a one-way ANOVA. Different letters show significant differences ($p<0.05$, $n=20$ for each level). Factor levels were colored: green for fresh and brown for dry conditions.

Diversity analyses at the species level showed that *Marchantia polymorpha* had significantly more unique metabolite features than the other species (Fig. 5b.). The homogeneity in the richness of relative abundances of features was significantly reduced in *M. polymorpha* when compared to the other species (Fig. 5d) due to significantly larger numbers of features in the dry condition.

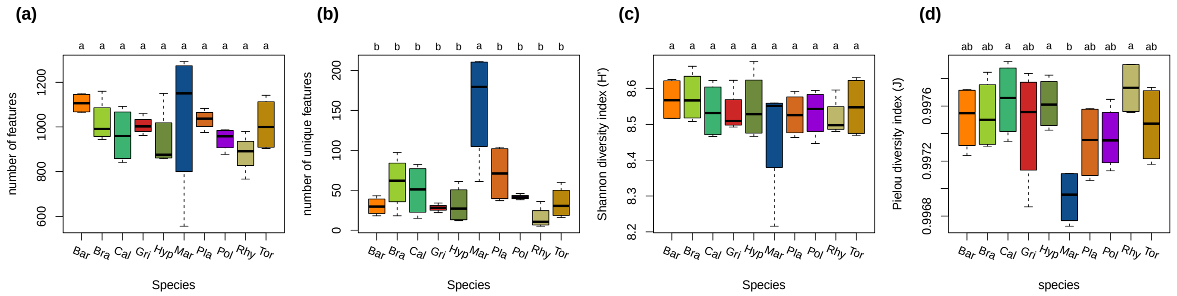

Figure S5. Diversity measures of metabolite features for the 10 bryophyte species. **(a)** Number of total features. **(b)** Number of unique features that were exclusively present in either of the species. **(c)** Shannon diversity indices (H') for the relative richness of features present in the species. **(d)** Pielou's evenness (J) for the homogeneity of the distribution of relative abundances in the species. Differences among groups (letters on the top of the plot) were calculated with performing the Tukey HSD post hoc on a one-way ANOVA. Different letters show significant differences ($p<0.05$, $n=4$ for each species). Species were colored: red and brown colors for acrocarpous species, green and yellow colors for pleurocarpous species, and blue color for liverworts.

S1.3 Comparison of in silico classification with metabolite fingerprinting

A total of 8465 MS/MS fragment spectra were classified in negative and positive ion modes. The MetFamily classifier was able to provide compound class annotations in 183 different compound classes.

First, differences between metabolite fingerprinting and *in silico* classification were assessed with variance partitioning and PCA (Fig. 6,7). While the herbarium conditions accounted for the majority of variation in the metabolite fingerprinting data in both ion modes (explained variation: 36% by herbarium conditions, 13% by the species), the amount of variation of the both study factors was similar in the classification matrix (explained variation: 22% by herbarium conditions, 25% by the species) (Fig. 6). Classification resulted in less separation of variation in both study factors (Fig. 7). *M. polymorpha* was more distinct with regard to compound classes than at the feature level (Fig. 7).

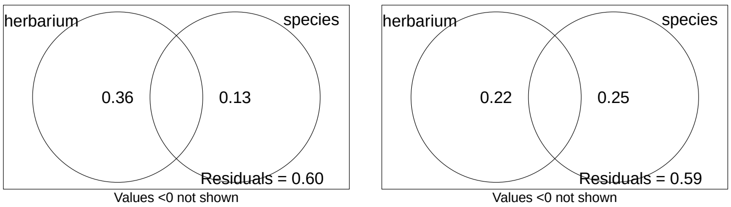

Figure S6. Variance partitioning for negative and positive on modes at the level of **(a)** metabolite fingerprinting using abundances of metabolite features, and **(b)** *in silico* classification resulting in compositional data of compound classes. The herbarium factor is shown in green, the species are shown in red. The value behind residuals is the amount of variation that was not explained by the two factors. n=80 samples.

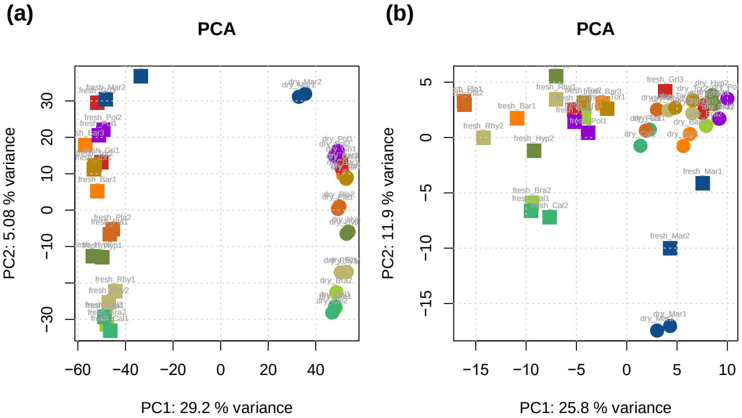

Figure S7. Principal Component Analysis for negative and positive ion modes at the level of **(a)** metabolite fingerprinting using a matrix with abundances of metabolite features, and **(b)** *in silico* classification using a matrix with compositional values of compounds belonging in the different compound classes. The scores were colored according to the ten species used in the study: red and brown colors for acrocarpous species, green and yellow colors for pleurocarpous species, and blue color for liverworts. A round shape of the scores represents samples of dry herbarium conditions and a square represents samples of fresh conditions.

S1.2. Exploring differences in the expression of metabolite families

Shown below is the ROC-curve for Fig. 4 in the main article.

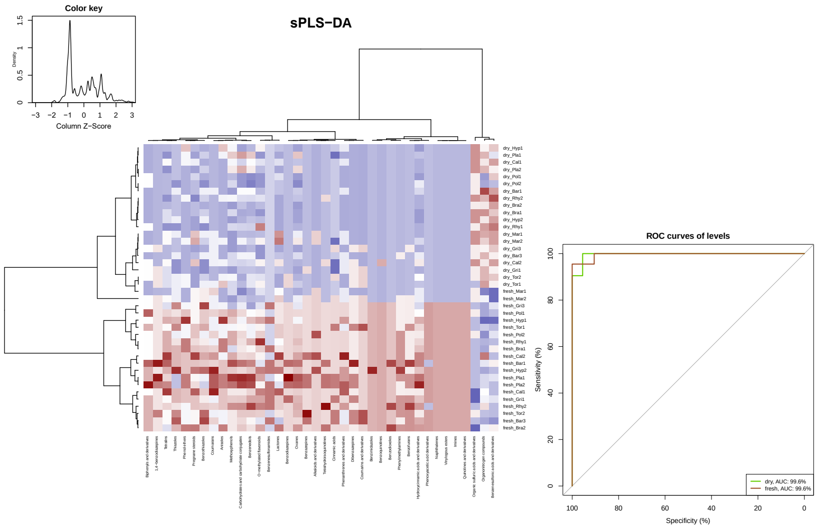

Figure S8. Results from the variable selection with sPLS-DA. **(a)** Heatmap of the selected variables by the sPLS-DA model using the compound classification table. Compound classes that were enriched are shown in red color and classes that were decreased are shown in blue color in the plots. **(b)** ROC curve. AUC=0.93.

Shown below is the AUC-PR-curve for Fig. 5 in the main article.

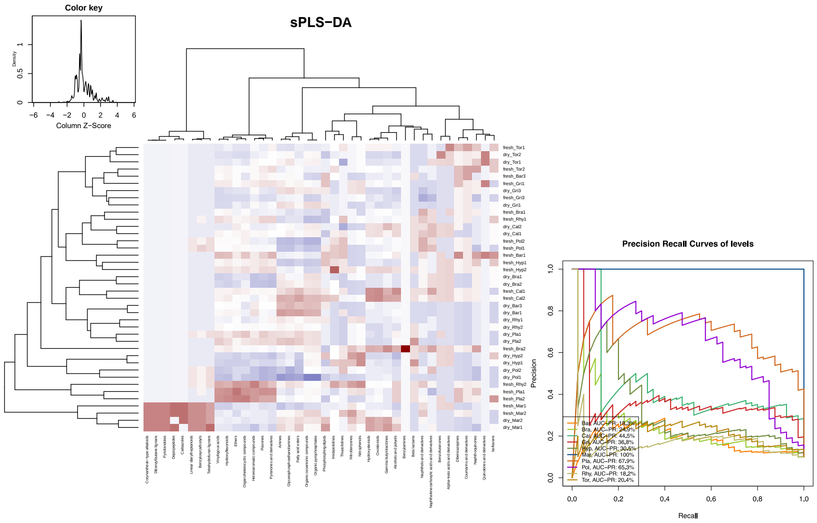

Figure S9. Differences in the composition of compounds belonging to compound classes at different levels of the chemical ontology with regard to the different species. **(a)** Heatmap of the selected variables by the sPLS-DA model. Compound classes that were enriched in the species are shown in red color and classes that were decreased are shown in blue color in the plots. **(c)** AUC-PR curve for the sPLS-DA model. Multiclass-AUC=0.458. AUC-PR=0.955.

Shown below are sunburst plots and the respective Fisher's exact tests showing differences at the level of metabolite families for the ten bryophyte species.

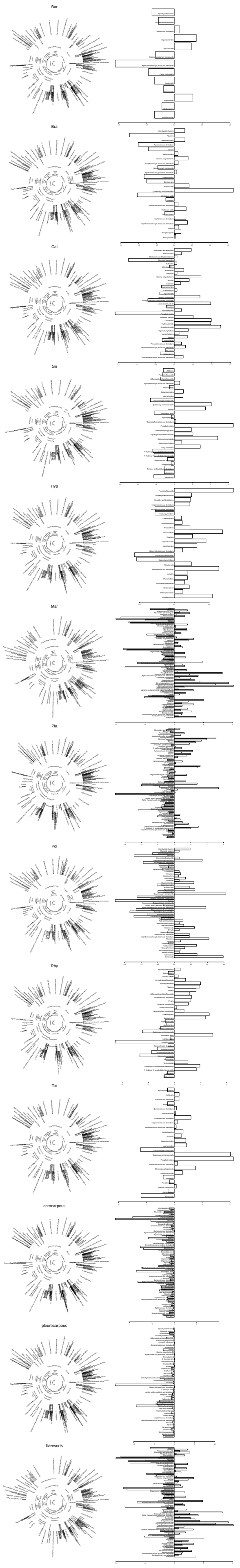
Bar
Bra
Cal
Gri
Hyp
Mar
Pla
Pol
Rhy
Tor
acrocarpous
pleurocarpous
liverworts

Figure S10. Differences in the composition of compounds belonging to compound classes at different levels of the chemical ontology with regard to the different species. The sunburst plot on the left showing the composition of compounds in the different species. Changes in relative frequencies of compound classes were determined using the Fisher's exact test and the frequencies are showed in the plot on the right.

S1.3. MetFamily classification model performances

Below we present tables showing the performances of the trained MetFamily models of negative and positive ion modes.

Table S1. List of classified compound classes in negative ion mode and the corresponding area under curve (AUC), area under precision–recall curve (AUC-PR), the true positive rate for a fixed false positive rate of 5% (FNR-FPR), and the true positive rate for a fixed false negative rate of 5% (TPR-FNR) determined by the MetFamily classifier in all of the bryophyte samples. The higher the AUC and AUC-PR values the better was the performance of the classifier. Generally, for the AUC-PR values above 0.5 are considered to be a good performance metric with imbalanced data. The lower the TPR-FPR the larger was for the probability that the classifier assigned chemical entities correctly to a compound class. The higher the TPR-FNR the larger was for the probability that the classifier assigned chemical entities correctly to a compound class.

**Negative ion mode: Compound class**	**CHEMONT ID**	**AUC**	**AUC-PR**	**TPR-FPR**	**TPR-FNR**
Alkaloids and derivatives	0000279	0,795	0,122	0,204	0,389
Corynanthean-type alkaloids	0002750	0,992	0,094	0,000	1,000
Benzenoids	0002448	0,774	0,777	0,284	0,309
Anthracenes	0000018	0,836	0,059	0,000	0,395
Anthracenecarboxylic acids and derivatives	0001911	0,829	0,022	0,000	0,250
Anthraquinones	0000151	0,870	0,045	0,000	0,417
Benzene and substituted derivatives	0002279	0,749	0,628	0,236	0,234
Anilides	0000285	0,720	0,027	0,000	0,267
Benzenesulfonic acids and derivatives	0000032	0,864	0,023	0,000	0,333
Benzoic acids and derivatives	0000176	0,772	0,150	0,288	0,158
Benzophenones	0000120	0,856	0,024	0,000	0,313
Benzoyl derivatives	0000321	0,759	0,190	0,232	0,164
Benzyl alcohols	0000033	0,762	0,006	0,000	0,000
Benzyloxycarbonyls	0001097	0,859	0,039	0,000	0,400
Biphenyls and derivatives	0000041	0,665	0,010	0,000	0,125
Butyrophenones	0000350	0,780	0,026	0,000	0,400
Diphenylethers	0004155	0,839	0,051	0,000	0,462
Diphenylmethanes	0000369	0,751	0,023	0,000	0,231
Halobenzenes	0000035	0,796	0,144	0,000	0,393
Methoxybenzenes	0004113	0,776	0,227	0,292	0,198
N-phenylureas	0001935	0,811	0,037	0,000	0,600
Nitrobenzenes	0000036	0,849	0,051	0,000	0,500
Phenoxy compounds	0004742	0,725	0,249	0,206	0,143
Phenylpropanes	0002811	0,602	0,011	0,000	0,077
Styrenes	0000037	0,773	0,171	0,213	0,250
Toluenes	0001091	0,659	0,041	0,000	0,206
Trifluoromethylbenzenes	0004551	0,811	0,040	0,000	0,429
Xylenes	0004208	0,697	0,012	0,000	0,143
Fluorenes	0000020	0,922	0,130	0,000	0,800
Naphthalenecarboxylic acids and derivatives	0001871	0,816	0,015	0,000	0,333

Naphthols and derivatives	0002441	0,741	0,029	0,000	0,385
Naphthoquinones	0000153	0,775	0,023	0,000	0,286
Phenanthrenes and derivatives	0000025	0,757	0,048	0,161	0,313
Phenol esters	0002319	0,810	0,017	0,000	0,188
Phenol ethers	0002341	0,752	0,367	0,279	0,222
Anisoles	0000138	0,781	0,358	0,310	0,268
1-hydroxy-2-unsubstituted benzenoids	0004646	0,786	0,571	0,258	0,309
1-hydroxy-4-unsubstituted benzenoids	0004647	0,800	0,491	0,265	0,345
4-alkoxyphenols	0004628	0,762	0,012	0,000	0,143
Benzenediols	0001286	0,796	0,228	0,217	0,232
Benzenetriols and derivatives	0001310	0,934	0,199	0,394	0,722
Cresols	0001272	0,801	0,054	0,128	0,320
Halophenols	0002320	0,854	0,125	0,292	0,545
Methoxyphenols	0000190	0,802	0,136	0,284	0,192
Nitrophenols	0000141	0,993	0,103	0,000	1,000
Tetralins	0000048	0,762	0,054	0,134	0,250
Lignans, neolignans and related compounds	0001392	0,828	0,084	0,443	0,309
Dibenzylbutane lignans	0001969	0,842	0,018	0,000	0,250
Tetrahydrofuran lignans	0001604	0,916	0,054	0,000	0,500
Lignan lactones	0001510	0,808	0,020	0,000	0,200
Eicosanoids	0000513	0,793	0,023	0,000	0,375
Fatty acid esters	0000324	0,859	0,626	0,423	0,456
Fatty acids and conjugates	0000262	0,713	0,142	0,200	0,183
Fatty acyl glycosides	0001766	0,843	0,232	0,392	0,442
Fatty acyl thioesters	0003327	0,997	0,186	0,000	1,000
Lineolic acids and derivatives	0000504	0,877	0,032	0,000	0,429
Glycerol ethers	0002391	0,943	0,213	0,761	0,587
Glycerol vinyl ethers	0003264	0,958	0,309	0,760	0,771
Glycerophospholipids	0000256	0,958	0,740	0,819	0,750
Glycerophosphocholines	0002213	0,962	0,520	0,824	0,798
Glycerophosphoethanolamines	0002215	0,959	0,477	0,859	0,703
Glycerophosphoglycerols	0002216	0,972	0,134	0,000	0,864
Glycerophosphoinositols	0002219	0,961	0,415	0,522	0,867
Glycerophosphoserines	0002218	0,993	0,268	0,000	1,000
Terpene glycosides	0002049	0,849	0,335	0,368	0,560
Phosphosphingolipids	0003392	0,991	0,189	0,000	1,000
Purine ribonucleotides	0001544	0,996	0,365	0,000	1,000
Pyrimidine nucleosides	0000480	0,884	0,102	0,000	0,583
Pyrimidine ribonucleotides	0002147	0,994	0,163	0,000	1,000
Ribonucleoside 3'-phosphates	0004404	0,990	0,196	0,000	1,000
Organic nitro compounds	0001152	0,813	0,066	0,000	0,471
Alpha-halocarboxylic acids and derivatives	0001548	0,973	0,125	0,000	0,667
Amino acids, peptides, and analogues	0000013	0,793	0,457	0,279	0,352
Carboxylic acid derivatives	0001093	0,694	0,612	0,209	0,097
Carboxylic acids	0001205	0,723	0,453	0,236	0,217
Dicarboxylic acids and derivatives	0000346	0,754	0,416	0,201	0,256
Alpha hydroxy acids and derivatives	0001359	0,773	0,046	0,154	0,250
Alpha-keto acids and derivatives	0001113	0,803	0,008	0,000	0,000
Organic carbonic acids and derivatives	0000364	0,804	0,109	0,247	0,381
Ureas	0000517	0,807	0,112	0,193	0,517

Phosphate esters	0000408	0,954	0,758	0,812	0,745
Organic sulfuric acids and derivatives	0000403	0,891	0,116	0,000	0,421
Depsipeptides	0001961	0,873	0,067	0,000	0,625
Thioesters	0001200	0,995	0,182	0,000	1,000
Vinylogous acids	0003889	0,803	0,414	0,259	0,392
Organonitrogen compounds	0000278	0,814	0,712	0,000	0,368
Amines	0002449	0,807	0,576	0,147	0,386
Quaternary ammonium salts	0000503	0,955	0,540	0,801	0,795
Sulfonylureas	0000490	0,883	0,021	0,000	0,333
Organic oxides	0003940	0,666	0,912	0,171	0,075
Organic oxoanionic compounds	0000463	0,961	0,339	0,000	0,933
Organic pyrophosphates	0001804	0,949	0,364	0,000	0,933
Organic oxoazanium compounds	0001508	0,856	0,072	0,000	0,531
Organooxygen compounds	0000323	0,808	0,993	0,326	0,321
Alcohols and polyols	0000129	0,725	0,770	0,218	0,272
Carbohydrates and carbohydrate conjugates	0000011	0,812	0,658	0,334	0,414
Carbonyl compounds	0001831	0,670	0,777	0,182	0,117
Ethers	0000254	0,739	0,730	0,218	0,279
Alkyl chlorides	0001026	0,725	0,013	0,000	0,250
Alkyl fluorides	0001027	0,856	0,104	0,277	0,523
Aryl bromides	0001029	0,896	0,182	0,000	0,708
Aryl chlorides	0001030	0,744	0,116	0,214	0,355
Aryl fluorides	0001031	0,771	0,038	0,000	0,333
Chlorohydrins	0002608	0,702	0,005	0,000	0,333
Organobromides	0001515	0,843	0,172	0,000	0,692
Organochlorides	0001516	0,796	0,155	0,203	0,356
Organofluorides	0001517	0,829	0,092	0,136	0,435
Vinyl chlorides	0002863	0,845	0,031	0,000	0,571
Organoheterocyclic compounds	0000002	0,734	0,810	0,209	0,280
Pyrazoles	0000087	0,743	0,018	0,000	0,250
Triazoles	0000099	0,860	0,045	0,000	0,571
Imidazolidines	0000250	0,836	0,049	0,000	0,667
Thiazolidines	0000226	0,751	0,065	0,000	0,667
Benzimidazoles	0000294	0,633	0,011	0,000	0,143
Benzodiazepines	0000295	0,863	0,014	0,000	0,300
1,4-benzodiazepines	0004097	0,843	0,010	0,000	0,250
Benzofuranones	0001578	0,836	0,008	0,000	0,333
Benzopyrans	0000123	0,817	0,481	0,267	0,410
1-benzopyrans	0003410	0,831	0,513	0,292	0,455
2-benzopyrans	0003411	0,724	0,016	0,000	0,200
Benzoxazines	0001396	0,752	0,005	0,000	0,333
Coumarans	0004189	0,726	0,043	0,157	0,156
Benzodiazines	0004789	0,804	0,034	0,000	0,444
Piperazines	0000189	0,571	0,007	0,000	0,000
Pyrazines	0000067	0,696	0,012	0,000	0,333
Pyrimidines and pyrimidine derivatives	0000075	0,860	0,325	0,438	0,530
1,3-dioxanes	0001314	0,826	0,019	0,000	0,357
Heteroaromatic compounds	0004144	0,705	0,423	0,203	0,178
Carbazoles	0000210	0,763	0,007	0,000	0,333
Indolecarboxylic acids and derivatives	0002013	0,888	0,011	0,000	0,250

Indolyl carboxylic acids and derivatives	0001290	0,900	0,035	0,000	0,429
Pyridoindoles	0001213	0,902	0,123	0,000	0,682
Isobenzofuranones	0003409	0,804	0,017	0,000	0,286
Isoindolines	0002496	0,818	0,035	0,000	0,375
Isoquinolones and derivatives	0002054	0,796	0,024	0,000	0,600
Beta lactams	0000162	0,965	0,086	0,000	0,600
Naphthofurans	0001634	0,732	0,029	0,000	0,176
Naphthopyranones	0001641	0,792	0,031	0,000	0,600
Oxacyclic compounds	0004140	0,763	0,774	0,230	0,336
Oxanes	0002012	0,799	0,624	0,313	0,373
Oxepanes	0001729	0,707	0,070	0,111	0,233
Pteridines and derivatives	0000109	0,913	0,023	0,000	0,400
Pyrans	0000086	0,765	0,353	0,252	0,245
Pyranones and derivatives	0000481	0,809	0,364	0,333	0,347
Hydropyridines	0002224	0,714	0,021	0,000	0,133
Methylpyridines	0004152	0,858	0,020	0,000	0,400
Pyridinecarboxylic acids and derivatives	0001322	0,771	0,041	0,000	0,308
Pyrrole carboxylic acids and derivatives	0002554	0,889	0,019	0,000	0,375
Pyrrolidones	0001158	0,628	0,015	0,000	0,333
Hydroquinolines	0001715	0,700	0,008	0,000	0,167
Quinolones and derivatives	0000056	0,753	0,012	0,000	0,250
Aminotriazines	0001693	0,735	0,013	0,000	0,333
Sulfenyl compounds	0003260	0,778	0,115	0,176	0,389
Sulfoxides	0000491	0,815	0,032	0,000	0,500
Aryl thioethers	0004631	0,759	0,009	0,000	0,375
2-arylbenzofuran flavonoids	0003405	0,806	0,017	0,000	0,286
Cinnamic acids and derivatives	0000476	0,827	0,172	0,278	0,285
Cinnamic acid amides	0002810	0,733	0,006	0,000	0,125
Cinnamic acid esters	0003480	0,864	0,176	0,419	0,336
Hydroxycinnamic acids and derivatives	0001391	0,868	0,212	0,291	0,478
Coumarin glycosides	0001743	0,880	0,028	0,000	0,300
Hydroxycoumarins	0002908	0,810	0,028	0,000	0,364
Depsides and depsidones	0001645	0,896	0,022	0,000	0,429
Linear diarylheptanoids	0002651	0,864	0,047	0,000	0,462
Biflavonoids and polyflavonoids	0001586	0,938	0,070	0,000	0,571
Flavans	0000337	0,871	0,171	0,351	0,547
Flavones	0001615	0,888	0,316	0,413	0,486
Flavonoid glycosides	0001111	0,900	0,362	0,593	0,574
Hydroxyflavonoids	0002991	0,881	0,434	0,383	0,533
O-methylated flavonoids	0002585	0,859	0,179	0,503	0,418
Isocoumarins and derivatives	0001890	0,716	0,005	0,000	0,000
Hydroxyisoflavonoids	0004154	0,809	0,068	0,238	0,409
Isoflav-2-enes	0002901	0,763	0,088	0,136	0,348
Isoflavans	0001612	0,803	0,057	0,000	0,438
Pyranoisoflavonoids	0003530	0,926	0,049	0,000	0,625
Linear 1,3-diarylpropanoids	0003467	0,801	0,034	0,000	0,294
Chalcones and dihydrochalcones	0001630	0,799	0,031	0,000	0,267
Cinnamylphenols	0003496	0,815	0,033	0,000	0,267
Macrolide lactams	0001900	0,855	0,023	0,000	0,500
Phenylpropanoic acids	0002551	0,794	0,048	0,203	0,283

Stilbenes	0000253	0,765	0,033	0,000	0,267
Tannins	0000238	0,877	0,074	0,000	0,500
Hydrolyzable tannins	0001710	0,885	0,032	0,000	0,286

Table S2. List of classified compound classes in positive ion mode and the corresponding area under curve (AUC), area under precision–recall curve (AUC-PR), the true positive rate for a fixed false positive rate of 5% (FNR-FPR), and the true positive rate for a fixed false negative rate of 5% (TPR-FNR) determined by the MetFamily classifier in all of the bryophyte samples. The higher the AUC and AUC-PR values the better was the performance of the classifier. Generally, for the AUC-PR values above 0.5 are considered to be a good performance metric with imbalanced data. The lower the TPR-FPR the larger was for the probability that the classifier assigned chemical entities correctly to a compound class. The higher the TPR-FNR the larger was for the probability that the classifier assigned chemical entities correctly to a compound class.

**Positive ion mode: Compound class**	**CHEMONT**	**AUC**	**AUC-PR**	**TPR-FPR**	**TPR-FNR**
Acetylides	0004478	0,836	0,030	0,000	0,200
Alkaloids and derivatives	0000279	0,802	0,192	0,065	0,286
Aniline and substituted anilines	0000284	0,779	0,196	0,097	0,278
Benzenesulfonamides	0000031	0,856	0,327	0,000	0,529
Benzophenones	0000120	0,779	0,029	0,000	0,200
Benzoyl derivatives	0000321	0,679	0,146	0,160	0,098
Benzylamines	0002673	0,728	0,020	0,000	0,000
Biphenyls and derivatives	0000041	0,752	0,045	0,000	0,250
Butyrophenones	0000350	0,733	0,011	0,000	0,000
Diphenylethers	0004155	0,756	0,021	0,000	0,200
Diphenylmethanes	0000369	0,733	0,049	0,000	0,154
Halobenzenes	0000035	0,799	0,342	0,216	0,262
Methoxybenzenes	0004113	0,757	0,179	0,126	0,207
Phenethylamines	0000186	0,738	0,058	0,000	0,154
Phenoxy compounds	0004742	0,680	0,218	0,081	0,122
Phenoxyacetic acid derivatives	0000417	0,880	0,054	0,000	0,333
Phenylmethylamines	0000185	0,671	0,011	0,000	0,167
Phenylpropanes	0002811	0,762	0,062	0,000	0,036
Styrenes	0000037	0,656	0,062	0,000	0,214
Trifluoromethylbenzenes	0004551	0,851	0,169	0,000	0,389
Xylenes	0004208	0,852	0,103	0,000	0,292
Naphthalenes	0000023	0,664	0,086	0,000	0,265
Phenanthrenes and derivatives	0000025	0,872	0,227	0,000	0,550
Phenol ethers	0002341	0,705	0,302	0,117	0,162
Anisoles	0000138	0,758	0,282	0,234	0,196
1-hydroxy-2-unsubstituted benzenoids	0004646	0,799	0,397	0,294	0,203
1-hydroxy-4-unsubstituted benzenoids	0004647	0,856	0,413	0,315	0,390
Benzenediols	0001286	0,882	0,307	0,000	0,526
Methoxyphenols	0000190	0,778	0,085	0,000	0,321
Tetralins	0000048	0,906	0,174	0,000	0,444
Lignans, neolignans and related compounds	0001392	0,959	0,722	0,000	0,750
Fatty acids and conjugates	0000262	0,831	0,170	0,351	0,357
Sesquiterpenoids	0001550	0,858	0,178	0,000	0,667
Hydroxysteroids	0001295	0,853	0,511	0,000	0,667
Oxosteroids	0001194	0,892	0,538	0,000	0,750
Pregnane steroids	0003569	0,901	0,595	0,000	0,667

Purine nucleosides	0000479	0,979	0,326	0,000	0,900
Purine ribonucleotides	0001544	0,992	0,518	0,000	1,000
Organic nitro compounds	0001152	0,761	0,092	0,000	0,222
Amino acids, peptides, and analogues	0000013	0,694	0,354	0,196	0,143
Carboxylic acid derivatives	0001093	0,636	0,370	0,130	0,110
Carboxylic acids	0001205	0,731	0,345	0,185	0,136
Dicarboxylic acids and derivatives	0000346	0,613	0,093	0,000	0,107
Phosphate esters	0000408	0,876	0,302	0,000	0,639
Organosulfonic acids and derivatives	0000270	0,797	0,291	0,191	0,362
Organic sulfuric acids and derivatives	0000403	0,842	0,064	0,000	0,400
Thiophosphoric acid esters	0001572	0,991	0,542	0,000	1,000
Hybrid peptides	0002010	0,866	0,342	0,000	0,500
Vinylogous acids	0003889	0,839	0,407	0,256	0,512
Vinylogous esters	0003891	0,750	0,029	0,000	0,333
Amines	0002449	0,675	0,578	0,151	0,170
Guanidines	0000375	0,896	0,262	0,000	0,429
Imines	0000117	0,673	0,100	0,000	0,167
Quaternary ammonium salts	0000503	0,668	0,102	0,000	0,357
Organic oxoanionic compounds	0000463	0,923	0,357	0,000	0,667
Organic pyrophosphates	0001804	0,923	0,357	0,000	0,667
Organic oxoazanium compounds	0001508	0,756	0,073	0,000	0,375
Alcohols and polyols	0000129	0,653	0,421	0,150	0,186
Carbohydrates and carbohydrate conjugates	0000011	0,889	0,594	0,368	0,567
Carbonyl compounds	0001831	0,671	0,585	0,215	0,143
Ethers	0000254	0,715	0,504	0,233	0,172
Alkyl chlorides	0001026	0,675	0,206	0,000	0,357
Alkyl fluorides	0001027	0,719	0,132	0,000	0,222
Aryl fluorides	0001031	0,787	0,221	0,000	0,333
Aryl iodides	0001032	0,991	0,700	0,000	1,000
Organofluorides	0001517	0,764	0,275	0,103	0,353
Organoiodides	0001518	0,884	0,388	0,000	0,750
Imidazoles	0000078	0,739	0,144	0,147	0,176
Pyrazoles	0000087	0,718	0,214	0,000	0,333
Thiazoles	0000095	0,745	0,081	0,000	0,300
Benzazepines	0000293	0,938	0,501	0,000	0,700
Dibenzazepines	0000320	0,936	0,419	0,000	0,625
Benzimidazoles	0000294	0,794	0,072	0,000	0,250
Benzodiazepines	0000295	0,895	0,283	0,000	0,500
1,4-benzodiazepines	0004097	0,918	0,348	0,000	0,600
Benzodioxoles	0000296	0,742	0,115	0,000	0,333
Benzofurans	0000301	0,783	0,056	0,000	0,167
Benzopyrans	0000123	0,903	0,512	0,562	0,522
1-benzopyrans	0003410	0,904	0,543	0,586	0,530
Benzothiazoles	0000311	0,672	0,249	0,000	0,400
Coumarans	0004189	0,793	0,270	0,000	0,500
Piperazines	0000189	0,750	0,176	0,000	0,353
Pyrimidines and pyrimidine derivatives	0000075	0,749	0,196	0,170	0,198
Heteroaromatic compounds	0004144	0,698	0,593	0,191	0,170
Imidazopyrimidines	0001797	0,894	0,383	0,000	0,632
Purines and purine derivatives	0000245	0,894	0,383	0,000	0,632

Indoles and derivatives	0000211	0,796	0,118	0,000	0,237
Lactones	0000050	0,782	0,133	0,128	0,224
Gamma butyrolactones	0001245	0,701	0,043	0,000	0,400
Oxacyclic compounds	0004140	0,729	0,456	0,232	0,149
Oxanes	0002012	0,891	0,483	0,410	0,589
Piperidines	0000195	0,757	0,173	0,149	0,224
Pyrans	0000086	0,857	0,387	0,308	0,370
Pyranones and derivatives	0000481	0,862	0,388	0,326	0,402
Hydroxypyridines	0004151	0,712	0,019	0,000	0,333
Pyridinecarboxylic acids and derivatives	0001322	0,771	0,226	0,000	0,346
Quinolines and derivatives	0001253	0,771	0,248	0,201	0,452
Benzoquinolines	0001908	0,837	0,125	0,000	0,571
Haloquinolines	0004403	0,853	0,530	0,000	0,625
Quinoline carboxylic acids	0002552	0,969	0,485	0,000	0,750
Quinolones and derivatives	0000056	0,896	0,283	0,000	0,667
Tetrahydrofurans	0002648	0,778	0,122	0,181	0,220
Tetrahydroisoquinolines	0002955	0,825	0,229	0,000	0,400
1,3,5-triazines	0004105	0,966	0,797	0,000	0,909
Aminotriazines	0001693	0,940	0,606	0,000	0,875
Cinnamic acids and derivatives	0000476	0,731	0,056	0,000	0,250
Cinnamic acids	0002504	0,900	0,024	0,000	0,333
Hydroxycinnamic acids and derivatives	0001391	0,727	0,051	0,000	0,313
Coumarins and derivatives	0000145	0,809	0,178	0,000	0,417
Flavans	0000337	0,888	0,094	0,000	0,429
Flavones	0001615	0,929	0,465	0,536	0,636
Flavonoid glycosides	0001111	0,985	0,816	0,837	0,897
Hydroxyflavonoids	0002991	0,948	0,546	0,767	0,679
O-methylated flavonoids	0002585	0,929	0,252	0,000	0,533
Isoflav-2-enes	0002901	0,859	0,036	0,000	0,200
Macrolactams	0000064	0,935	0,671	0,000	0,667
Macrolides and analogues	0000147	0,911	0,201	0,000	0,400
Phenylpropanoic acids	0002551	0,876	0,022	0,000	0,167
Stilbenes	0000253	0,829	0,028	0,000	0,250

S1.4. Quality Control

Below we present diagnostic plots to assess the analytical and technical quality control.

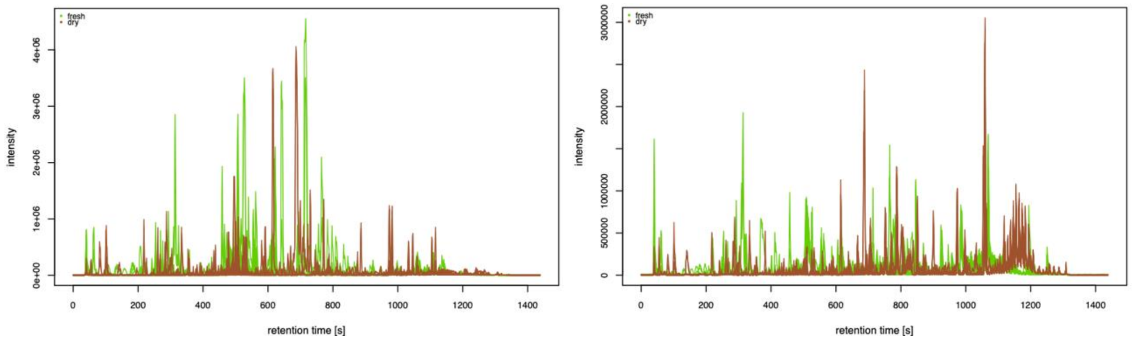

Figure S11. Raw chromatograms of metabolite profiles of the 40 bryophyte samples acquired in **(a)** negative mode, and **(b)** positive ion mode. Chromatograms were colored: green for fresh and brown for dry conditions. n=20.

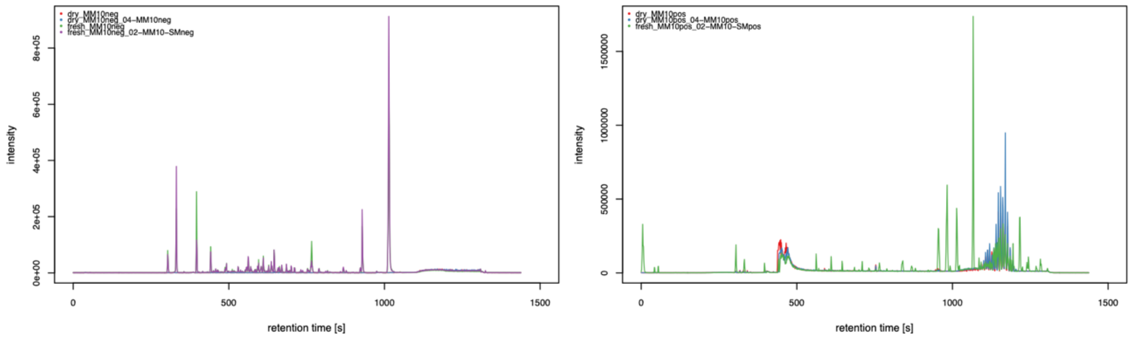

Figure S12. Raw chromatograms of sets of the MM10 profiles to assess the performance of the technical setup. **(a)** Raw chromatograms acquired in negative mode, and **(b)** Raw chromatograms acquired in positive ion mode.

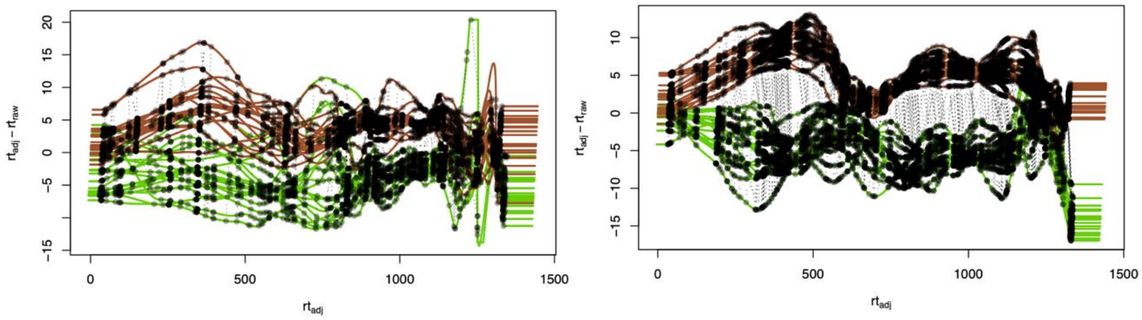

Figure S13. Quality control plots to assess shifts in retention time (RT) in the 40 metabolite profiles of the bryophytes. **(a)** Deviations in retention times for profiles acquired in negative mode, and **(b)** Deviations in retention times for profiles acquired in positive ion mode. Deviations were colored: green for fresh and brown for dry conditions. n=20.

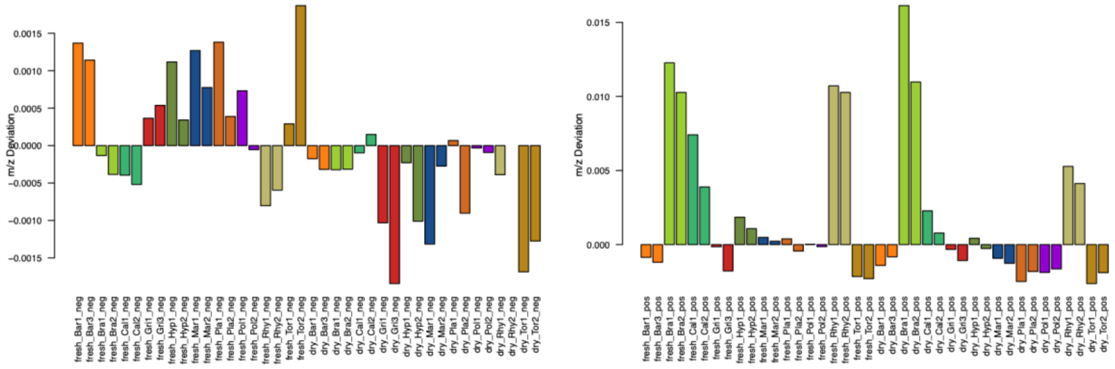

Figure S14. Quality control plots to assess shifts in mass-to-charge ratios (m/z) in the 40 metabolite profiles of the bryophytes. **(a)** Median m/z deviation for each sample acquired in negative mode, and **(b)** Median m/z deviation for each sample acquired in positive ion mode. The samples were colored according to the 10 species used in the study: red and brown colors for acrocarpous species, green and yellow colors for pleurocarpous species, and blue color for liverworts.

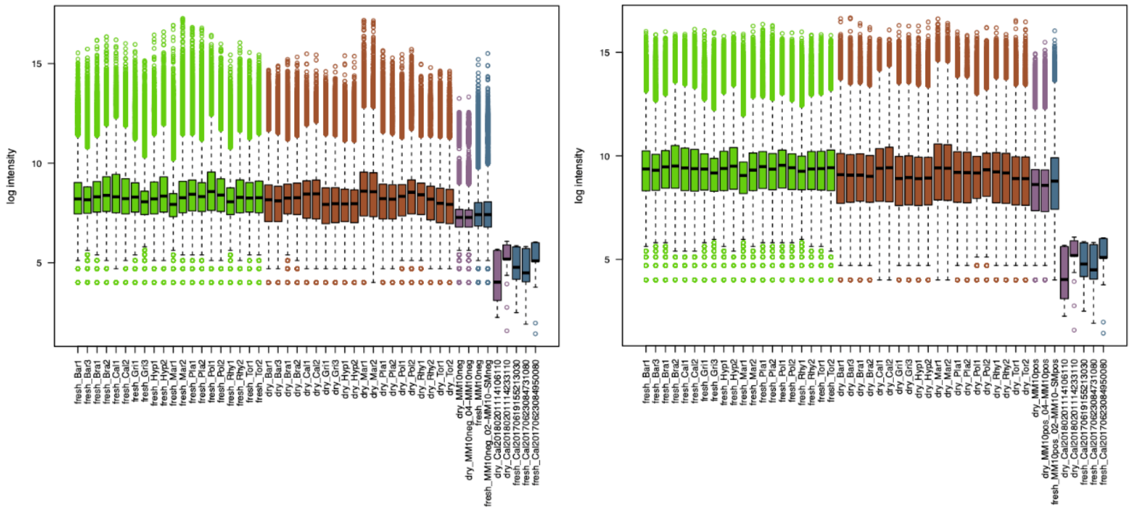

Figure S15. Quality control plots to assess total ion currents (TIC) in the 40 metabolite profiles of the bryophytes and the quality control samples. **(a)** Total ion current for profiles acquired in negative mode, and **(b)** Total ion current for profiles acquired in positive ion mode. TICs were colored: green for samples acquired under fresh conditions and brown for samples acquired under dry conditions. QC samples were colored: dark violet for QC samples under dry and dark blue for QC samples under fresh conditions. Shown right next to the 40 bryophyte samples are the MM10 quality control samples and the blanks.
